# The 5′UTR in human adenoviruses: leader diversity in late gene expression

**DOI:** 10.1038/s41598-017-00747-y

**Published:** 2017-04-04

**Authors:** Mirja Ramke, Jeong Yoon Lee, David W. Dyer, Donald Seto, Jaya Rajaiya, James Chodosh

**Affiliations:** 1 Howe Laboratory, Massachusetts Eye and Ear Infirmary, Department of Ophthalmology, Harvard Medical School, 243 Charles Street, Boston, Massachusetts USA; 2 Department of Microbiology and Immunology, University of Oklahoma Health Sciences Center, Oklahoma City, Oklahoma USA; 3 Bioinformatics and Computational Biology Program, School of Systems Biology, George Mason University, Manassas, Virginia USA

## Abstract

Human adenoviruses (HAdVs) shut down host cellular cap-dependent mRNA translation while initiating the translation of viral late mRNAs in a cap-independent manner. HAdV 5′ untranslated regions (5′UTRs) are crucial for cap-independent initiation, and influence mRNA localization and stability. However, HAdV translational regulation remains relatively uncharacterized. The HAdV tripartite leader (TPL), composed of three introns (TPL 1–3), is critical to the translation of HAdV late mRNA. Herein, we annotated and analyzed 72 HAdV genotypes for the HAdV TPL and another previously described leader, the i-leader. Using HAdV species D, type 37 (HAdV-D37), we show by reverse transcription PCR and Sanger sequencing that mRNAs of the HAdV-D37 E3 transcription unit are spliced to the TPL. We also identified a polycistronic mRNA for RID-α and RID-β. Analysis of the i-leader revealed a potential open reading frame within the leader sequence and the termination of this potential protein in TPL3. A potential new leader embedded within the E3 region was also detected and tentatively named the j-leader. These results suggest an underappreciated complexity of post-transcriptional regulation, and the importance of HAdV 5′UTRs for precisely coordinated viral protein expression along the path from genotype to phenotype.

## Introduction

Human adenoviruses (HAdVs) are double stranded, non-enveloped DNA viruses with 72 types currently deposited in GenBank, distributed into seven species (A-G), and are associated with a broad spectrum of diseases^1–3^. All HAdV genomes share a similar organization, albeit with differences in genome length and gene occurrence. Early, intermediate, and late genes are expressed in a step-wise manner and named in accordance with the timing of their transcription and translation during the HAdV replication cycle^4^. Early genes are the first to be transcribed and translated, and once a specific threshold of early proteins is reached, virus genome replication is initiated^5^. This in turn activates the major late promoter (MLP), and indicates the beginning of late viral gene expression, with transcription of the late genes located in the major late transcription unit^6–8^. Notably, the E3 genes, known for their ability to modulate host immune responses, are located in the major late transcription unit^9, 10^. All late genes are initially transcribed as a single pre-mRNA strand, followed by extensive and complex alternative splicing into multiple mRNAs. This alternative splicing, discovered in 1977 in HAdV-C2^11, 12^, leads to mature mRNAs which possess leader sequence from the 5′ untranslated region (UTR) of pre-mRNA. The most common leader found in late-expressed mRNAs is the tripartite leader (TPL), consisting of leaders 1, 2, and 3^13–15^. Four additional leaders, namely the i-leader, and the x, y, and z-leaders, were also characterized in HAdV-C2, and in -C5^16, 17^. Besides their annotation in viruses within HAdV-C, TPLs 1–3 have been annotated but not experimentally studied in HAdV-A12, HAdV-B11p, HAdV-B55, and HAdV-D9. The i-leader was annotated in HAdV-E4 and HAdV-F40. In contrast, the x, y, and z-leaders were described only in HAdV-C. The latter leaders were shown to play a particularly important role in the alternatively splicing of the fiber gene^4^, where their presence allows the fiber mRNA to accumulate more efficiently, as compared to the other late mRNAs^16^.

Protein translation in eukaryotic cells typically begins with binding of the 5′cap to the eIF4F complex^18^, followed by recruitment to the 43S preinitiation complex, resulting in translation initiation. HAdVs, like many other viruses, inhibit initiation of host cell 5′ cap-dependent mRNA translation^19^, in favor of viral late gene expression in a cap-independent manner, and requiring the viral 5′UTR. Complementary binding sites within viral 5′UTRs to the 18S ribosomal RNA allow direct recruitment of the ribosomal complex to the mRNA without a cap-recruitment complex^20–22^. Aside from initiation of translation, eukaryotic 5′UTRs perform other functions, including the regulation of mRNA stability and mRNA nuclear export; each impacts protein expression. Secondary structure, 18S RNA complementarity, binding sites for RNA binding proteins, u-motifs, and uAUGs and uORFs have been reported as important regulatory elements of 5′UTRs, but GC content and 5′UTR length also contribute^23, 24^. However, the interplay between these elements, and their relative importance to late gene expression, are not fully understood.

Despite the 5′UTR’s significance in translation initiation and post-transcriptional regulation, a comprehensive analysis of the HAdV 5′UTRs has not been performed, and only 6 out of 72 HAdV types available in GenBank have the TPL annotated. Additionally, detailed analysis of the 5′UTR of HAdV-D, the species with the most characterized genotypes, is lacking. We annotated the TPL sequences in all 72 HAdV genotypes, and performed RT-PCR and Sanger sequencing to characterize late mRNAs of the clinically important virus, HAdV-D37. We present herein the first comprehensive analysis of the 5′UTRs of HAdV types.

## Results

### Genome structure and leader arrangement among human adenovirus species is similar

**Figure 1:**
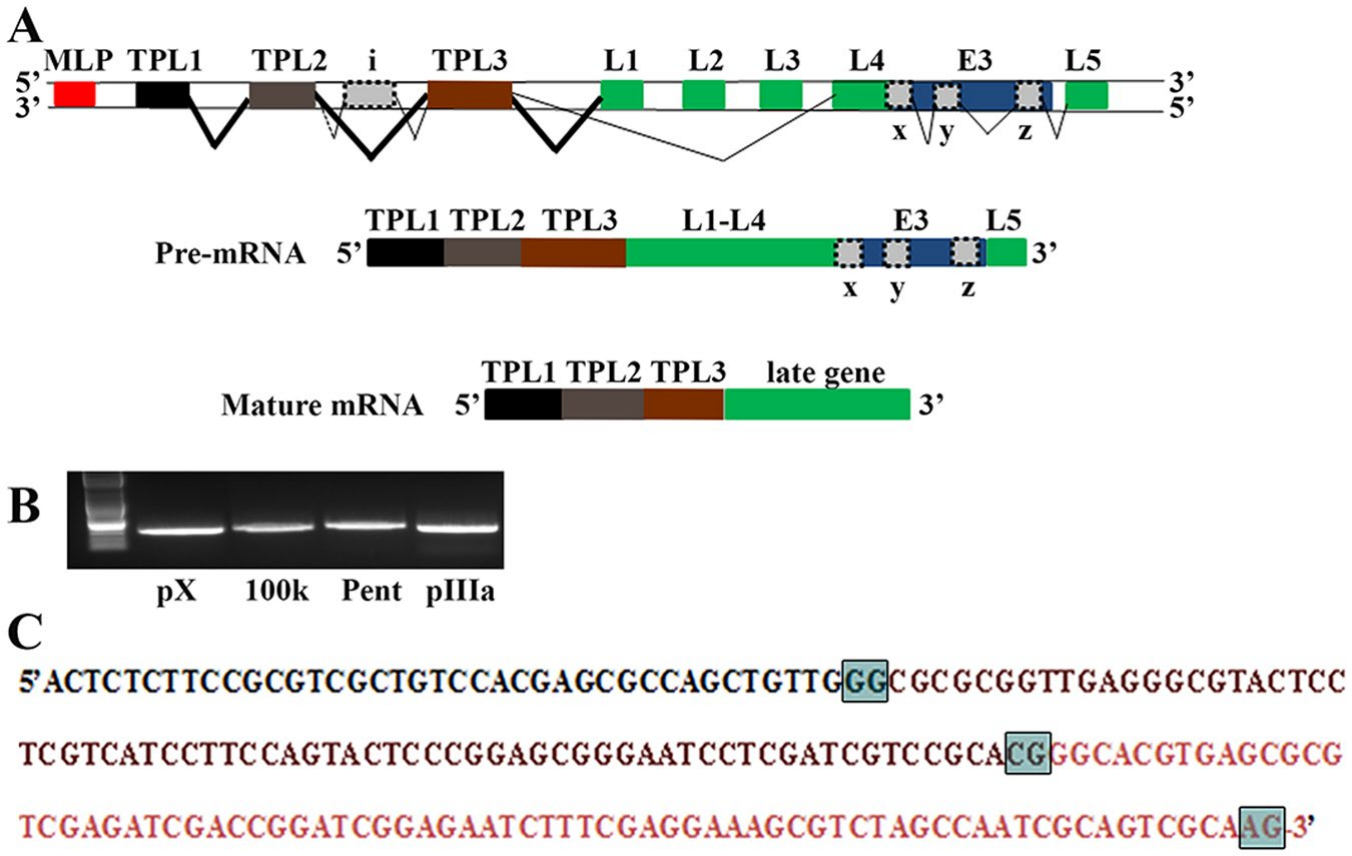
Organization of the human adenovirus late transcription unit. (**A**) Schematic of the major late transcription unit of HAdV-C2^adapted from 4^, chosen because of prior experimental evidence for the shown leader sequences. Major late promoter (MLP: red; Late gene family L1-L5: green; E3 transcription unit: blue; tripartite leader (TPL) 1, 2, 3, and i, x, y, and z-leaders: grey). The thickness of the angled lines indicates the approximate abundance of the splice events in the referenced paper. Common splicing events between TPL3 and L3, y, and L5 were omitted for simplicity. (**B**) To examine splicing of the tripartite leader of HAdV-D37 during natural infection, human A549 cells were infected for 24 hrs. DNA was removed by DNase treatment. cDNA was amplified by using a forward primer for HAdV-D37 TPL1 and a reverse primer within the following late genes: protein X (pX), 100 kDa, penton base (Pent), and pIIIa. Primers were chosen to elicit similarly sized bands to facilitate subsequent sequencing. In each case, TPL1-3 was found spliced to the late gene 5′ end. (**C**) TPL1-3 as Sanger sequenced from and common to each gel purified transcript in (**B**). (TPL1: black; TPL2: purple, TPL3: pink; and splice sites: boxed in grey).

In HAdV-C2, the most common mature mRNA leader arrangement was shown to be TPL1-TPL2-TPL3-late gene^4^. A schematic based on HAdV-C, showing the relative locations of the major late promoter (MLP) and the major late transcription unit (consisting of the late genes (L1-L5), the E3 region, the tripartite leaders 1, 2, and 3, and the less characterized leaders i, x, y, and z), is shown in Fig. 1A. We also annotated the TPL1–3 for all 72 HAdV genotypes then in GenBank (Supplemental Table 1), using MEGA 6.06 (www.megasoftware.net), and confirmed the data by splice site prediction analysis by using the “Alternative Splice Site Predictor” software (ASSP, www.wangcomputing.com, Seville, Spain). In HAdV-F40, and -D9, we obtained slightly different TPL annotation results than in GenBank, as shown in the Table. To confirm experimentally the presence and splicing of the tripartite leader in transcripts during infection by HAdV-D37, mRNA from infected human A549 cells was harvested at 12 and 24 hours post infection (hpi), and after RT-PCR with forward primers from TPL1 and reverse primers from select late genes (Fig. 1B), the cDNAs were sequenced and annotated (Fig. 1C). In each case, TPL1–3 was spliced to the 5′ end of the late gene. Similar data was obtained from both time points post infection; the data shown are from the 24 hpi time-point. To eliminate the possibility of cell type-specific effects, we also confirmed our results in human corneal fibroblasts and epithelial cells (data not shown), natural target cells for HAdV-D37 infection^7^.

**Figure 2:**
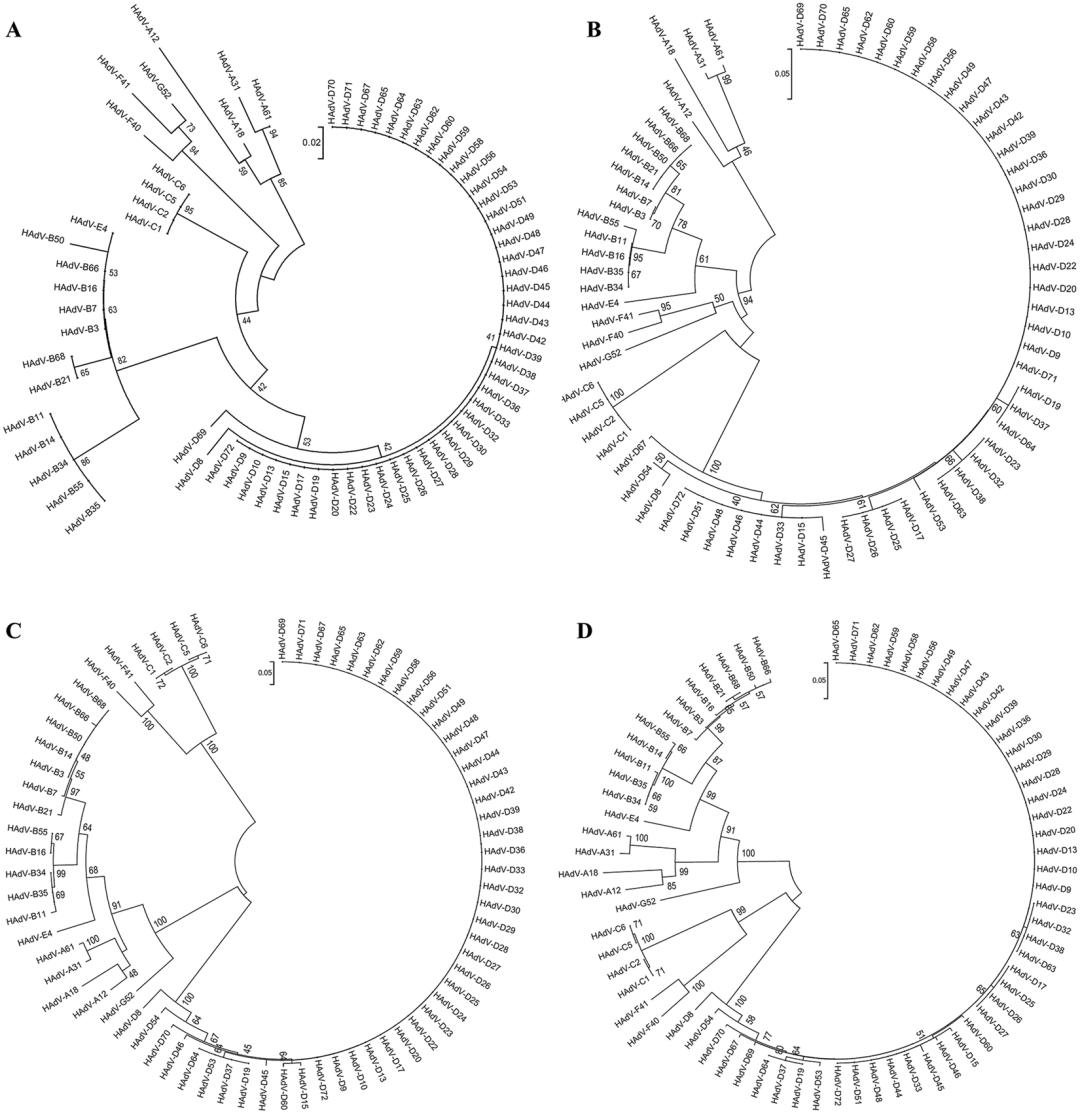
Phylogenetic analysis of the HAdV tripartite leader in HAdV. The TPL1-3 of each typed HAdV was annotated by blast, aligned using MEGA 6.06, and the splice sites predicted (http://wangcomputing.com/assp/). Phylogenetic neighbor-joining trees, bootstrap-confirmed (1000 replicates) were constructed for (**A**) TPL1, (**B**) TPL2, (**C**) TPL3, and (**D**) TPL1-3.

To determine relative diversity in TPL1–3 across HAdV genotypes and species, bootstrapped, neighbor-joining trees with 1,000 replicates of TPL1, TPL2, TPL3, and TPL1–3 (MEGA 6.0.6), for all known 72 types were then constructed, revealing relative nucleotide conservation within species, but diversity between species (Fig. 2A–D). These data are consistent with other relatively conserved areas of the genome^25^, and suggest that HAdV species could be differentiated from one another by TPL analysis alone.

### Representative TPL from each species show diversity in GC content and secondary structure

To further analyze the differences in TPLs between HAdV species, one representative virus from each species (including one virus from each of the two HAdV-B sub-species) was chosen. By analysis with Clustal Omega (http://www.ebi.ac.uk/Tools/msa/clustalo)^26^, we found identities for TPL1–3 ranging from 67% (between A12 and F40) to 76.50% (between A12 and B3) (Table 1). The identity comparisons in percentages between representative viruses of each species for TPL1, TPL2, and TPL3 individually are shown in Supplemental Table 2. The lengths (numbers of nucleotides) of TPL1 and TPL2 were found to be conserved throughout all species with 41 and 72 nucleotides, respectively (Table 2). In contrast, TPL3 exhibited length polymorphisms that ranged from 75 nucleotides (species D), to 87 (species A, B1, B2, E, and G), to 90 (species C, and F). GC-rich regions of genomes are thought to confer evolutionary stability^27^, and act in 5′UTRs^23^ to affect mRNA translation^28^. We analyzed the GC content of TPL1–3 for each virus (Supplemental Tables 3–5) and also compared the GC content for one representative virus of each HAdV species to the GC content of the respective full genome (Table 3). Notably, all HAdV species TPL showed higher GC content than for the total (parent) genome; the TPL1–3 of HAdV-A12 and -D37 had GC contents about 10% higher than the average whole genome GC content, while all others had a GC proportion within 10% of the whole genome GC.

**Table 1:** Percent identity matrix for tripartite leader in representative HAdV types across species.

Virus species and type	HAdV-A12	HAdV-B3	HAdV-B11	HAdV-C2	HAdV-D37	HAdV-E4	HAdV-F40	HAdV-G52
HAdV-A12	100.00							
HAdV-B3	76.50	100.00						
HAdV-B11	74.00	91.50	100.00					
HAdV-C2	72.50	79.50	77.50	100.00				
HAdV-D37	70.81	77.84	75.68	78.19	100.00			
HAdV-E4	74.50	89.00	85.00	78.00	75.14	100.00		
HAdV-F40	67.00	72.00	72.50	68.47	77.66	70.50	100.00	
HAdV-G52	72.00	78.00	76.00	75.50	77.30	74.00	78.50	100.00

Note HAdV-B has two sub-species.

**Table 2:** Tripartite leader sequence lengths in nucleotides by HAdV species.

HAdV species	TPL1	TPL2	TPL3	TPL 1–3
HAdV-A	41	72	87	200
HAdV-B:1	41	72	87	200
HAdV-B:2	41	72	87	200
HAdV-C	41	72	90	203
HAdV-D	41	72	75	188
HAdV-E	41	72	87	200
HAdV-F	41	72	90	203
HAdV-G	41	72	87	200

**Table 3:** Tripartite leader GC content by percent in representative HAdV types across species.

Virus species and type	TPL1	TPL2	TPL3	TPL 1–3	Whole genome
HAdV-A12	53.7	47.2	54	51.5	46.5
HAdV-B3	63.4	51.4	52.9	54.5	51.1
HAdV-B11	58.5	51.4	50.6	52.5	48.9
HAdV-C2	63.4	58.3	60.0	60.1	55.2
HAdV-D37	65.9	65.3	58.7	62.8	56.6
HAdV-E4	63.4	61.1	57.5	60.0	57.7
HAdV-F40	58.5	59.7	52.2	56.2	51.2
HAdV-G52	58.5	54.2	55.2	55.5	55.1

Secondary structures of 5′UTRs have been demonstrated as an important element in translation regulation and mRNA stability^23, 28^. In particular, it has been shown that the minimum free energy (MFE) of the secondary structure and the distance of hairpin loops to the ATG are critical features for translation efficiency^28^. To examine whether the most common leaders, TPL1–3, of different species form similar minimum free energy secondary structures, and thus may possess similar translation efficiencies, we predicted the secondary structure of one representative virus of each species and two representatives for species B: one for subspecies B1 and one for B2, based on our TPL phylogeny results above. Using the mFold RNA secondary structure prediction software^29^, we chose the structure with the lowest minimum free energy (the structure that is most likely to form in nature), and found differences between species, with minimum free energies ranging from dG = −52.36 in HAdV-A12 to dG = −70.05 in HAdV-D37 (Supplemental Fig. 1). These data suggest there may be corresponding differences in the translation efficiency of TPLs between species, but overall the structures appeared similar. We also assessed 18S complementarity and found nearly identical results across species (Supplemental Fig. 2).

### The i-leader includes a potential ORF and terminates in TPL3

**Figure 3:**
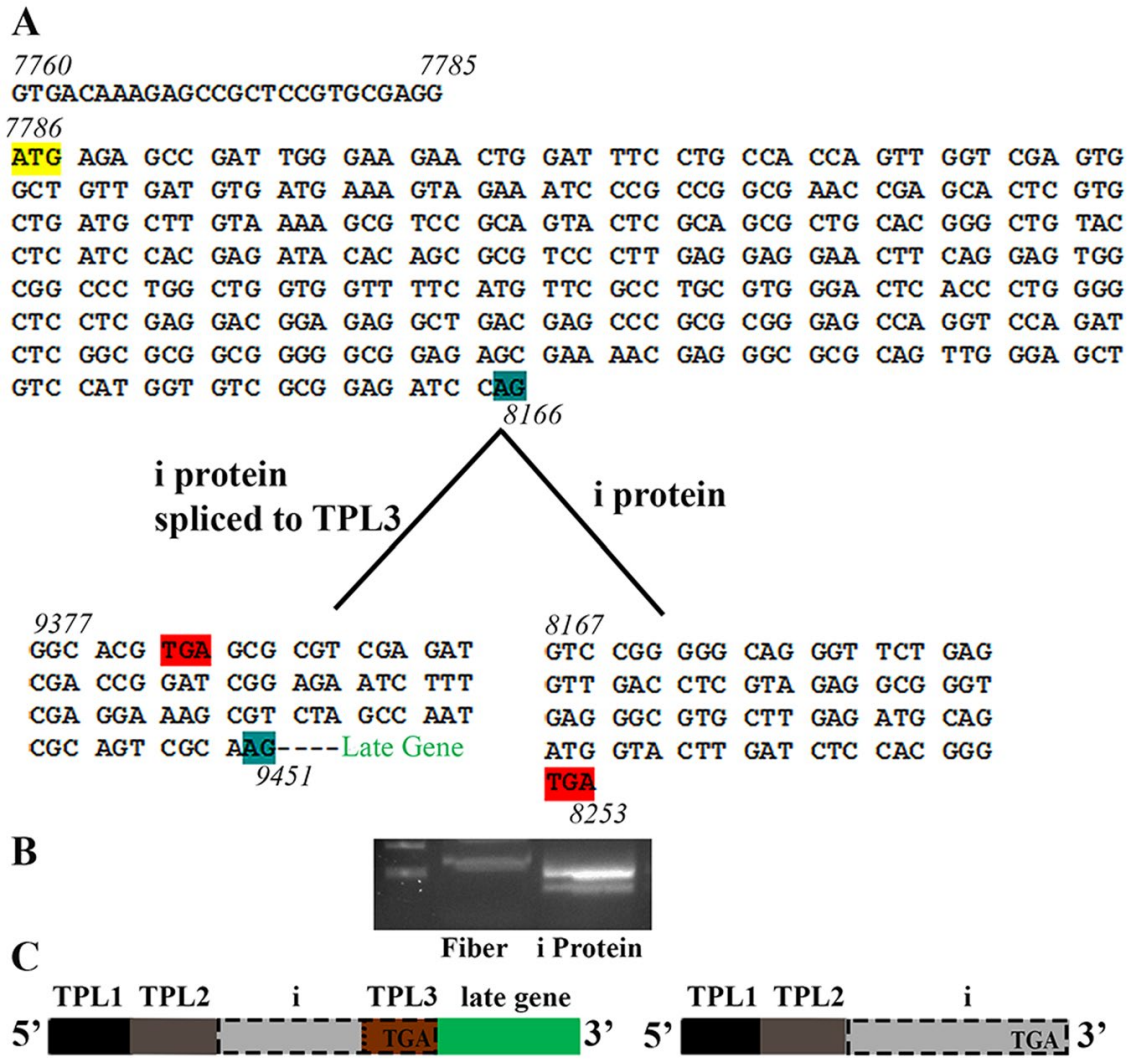
The putative i-leader protein terminates in the TPL3 of HAdV-D37. (**A**) Nucleotide sequence, (**B**) RT-PCR gel photomicrograph, and (**C**) schematic for i-leader spliced to TPL3 of 5′UTR for fiber, and in the putative i-protein mRNA, in which the 5′UTR is TPL1-2. RT-PCR as shown was performed with forward primer from TPL1 and reverse primers either from within fiber gene or the end of the predicted i-protein. Sequencing of gel purified transcripts revealed two splice variants of the putative i-leader mRNA, as shown. The putative i-protein mRNA is preceded by a 26 nucleotide 5′UTR prior to the start site (yellow) of an ORF (7786) for the potential coding region that would terminate (red) either within TPL3 (as shown for fiber gene), or at nucleotide 8253, the latter coding for the putative i-protein.

The i-leader was previously described for HAdV-C2 and -C5^4, 16, 30, 31^. It has been shown that the HAdV-C5 i-leader encodes a 13.6 kDa protein^31^. The presence of the i-leader in the L1 52/55 kDa mRNA reduces mRNA half-life^32^ while truncation of the i-leader improves oncolytic adenovirus efficacy^33, 34^. In HAdV-C2, three different splice variants of the i-leader have been described^30^. To examine for the existence of an i-leader in HAdV-D37 mRNAs, forward PCR primers for TPL1 and i-leader and reverse primers for L1 52/55 K, L1 pIIIa, L5 fiber, and the i-leader were designed in the OligoAnalyzer Tool from Integrated DNA Technology (IDT, Coralville, IA), and the resulting RT-PCR products gel purified and sequenced. We confirmed presence of the i-leader spliced in some but not all mature mRNAs, resulting variably in either TPL1-2-i-3-late gene or TPL1-2-i configurations (Fig. 3). In contrast to other leader sequences, the i-leader also contains a potential ORF of two possible lengths. A potential i-protein was described previously in HAdV-C2 and -C5^30, 31, 35, 36^, and was previously annotated in HAdV-E4 in GenBank. Our group also previously predicted a hypothetical 16.57 kDa with an ORF located in the i-leader^7^. By RT-PCR with subsequent sequencing, in TPL1-2-i-3-late gene transcripts, the i-leader terminates (TAG) within TPL3 (Fig. 3B,C). In the 1-2-i transcripts, the i-leader transcript is not spliced before reaching the stop codon, and terminates within the i-leader sequence itself. By splice site and codon analyses of one representative virus for each species, we found the splice site acceptor boundary site 26 nt upstream of the ATG, except for HAdV-G52 (15 nt upstream), and a potential termination codon in TPL3 (Supplemental Table 6). These findings suggest the presence of the i-leader in all HAdV species, and additionally lend credence to the possibility of an i protein.

### The 5′UTR within the E3 region of HAdV-D37 is diverse

**Figure 4:**
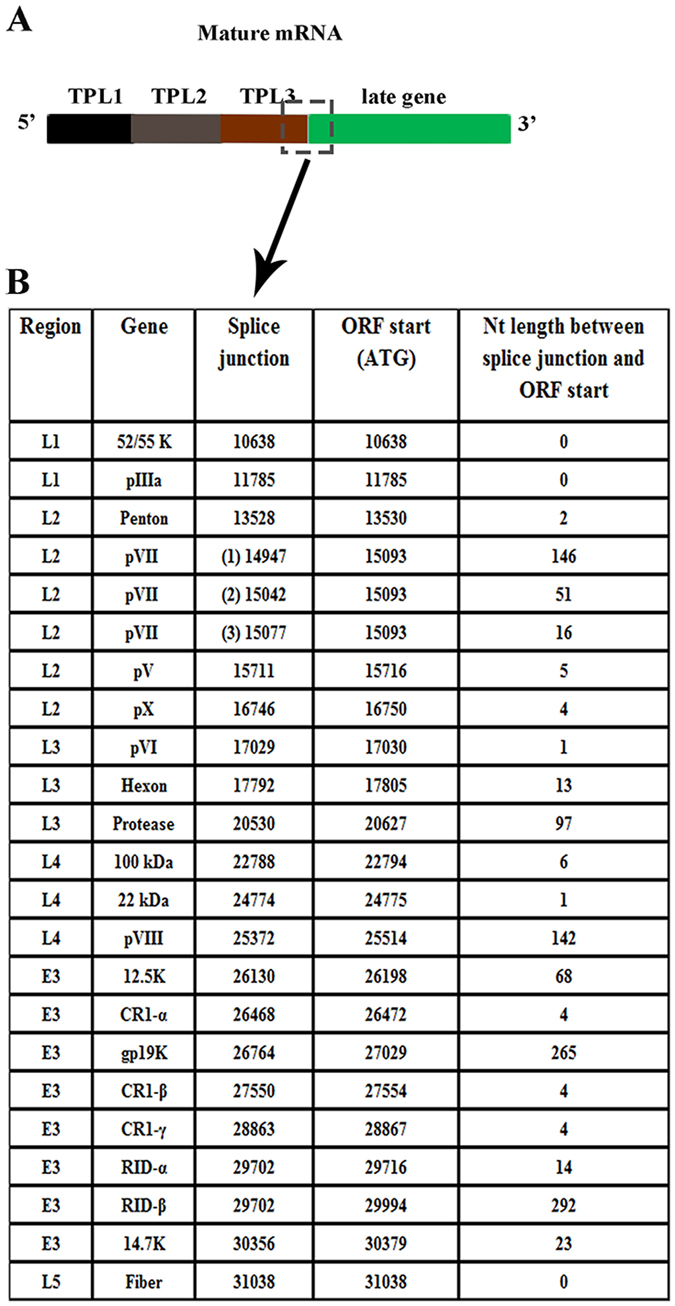
Splice sites for junction between tripartite leader 3 and genes transcribed by the major late promoter in HAdV-D37. (**A**) Schematic for splice site junctions in mature mRNA of genes under the control of the major late promoter (dashed box: splice site). (**B**) Table showing the genome region, gene name, splice site, start site, and leader length for each gene transcribed under the control of the major late promoter in HAdV-D37. A549 cells were infected with HAdV-D37 at MOI of 10, mRNA was harvested at 24 hpi, and DNA removed by treatment with DNase. cDNA was generated, and PCR performed with forward primer for TPL1 and a reverse primer for each late and E3 gene. PCR products were gel purified and sequenced. Notably, mRNAs for the E3 genes RID-α and RID-β showed the same splice site, resulting in one mRNA for both genes, consistent with a polycistronic mRNA.

The 5′UTR is defined as the noncoding leader region upstream of an AUG. In HAdV late genes, the 5′UTR is typically thought to be the TPL, disregarding the contribution of alternatively spliced leader sequence between the acceptor splice site and the AUG, with potential impact on mRNA stability, nuclear export, and secondary structure. Further, the E3 gene region is located within the major late transcription unit. To examine which HAdV-species D E3 genes are spliced to the TPL, we infected A549 cells with HAdV-D37, performed RT-PCR with 5′ primers from TPL1, and 3′ primers from the gene of interest, and sequenced the PCR products with attention to the presence of TPL1-3, the splice acceptor sites, and the number of nucleotides between the splice site and the start AUG (Fig. 4). We found TPL1-3 in all the late and E3 gene mRNAs. Notably, we found three possible splice sites in pVII, ranging from 16 to 146 nucleotides between the start AUG and the splice acceptor side. Also, it appears that RID-α, and RID-β share the same splice acceptor side, resulting in a polycistronic mRNA.

### HAdV-D37 mRNA contains a previously unknown leader, embedded in the E3 CR1-α gene

**Figure 5:**
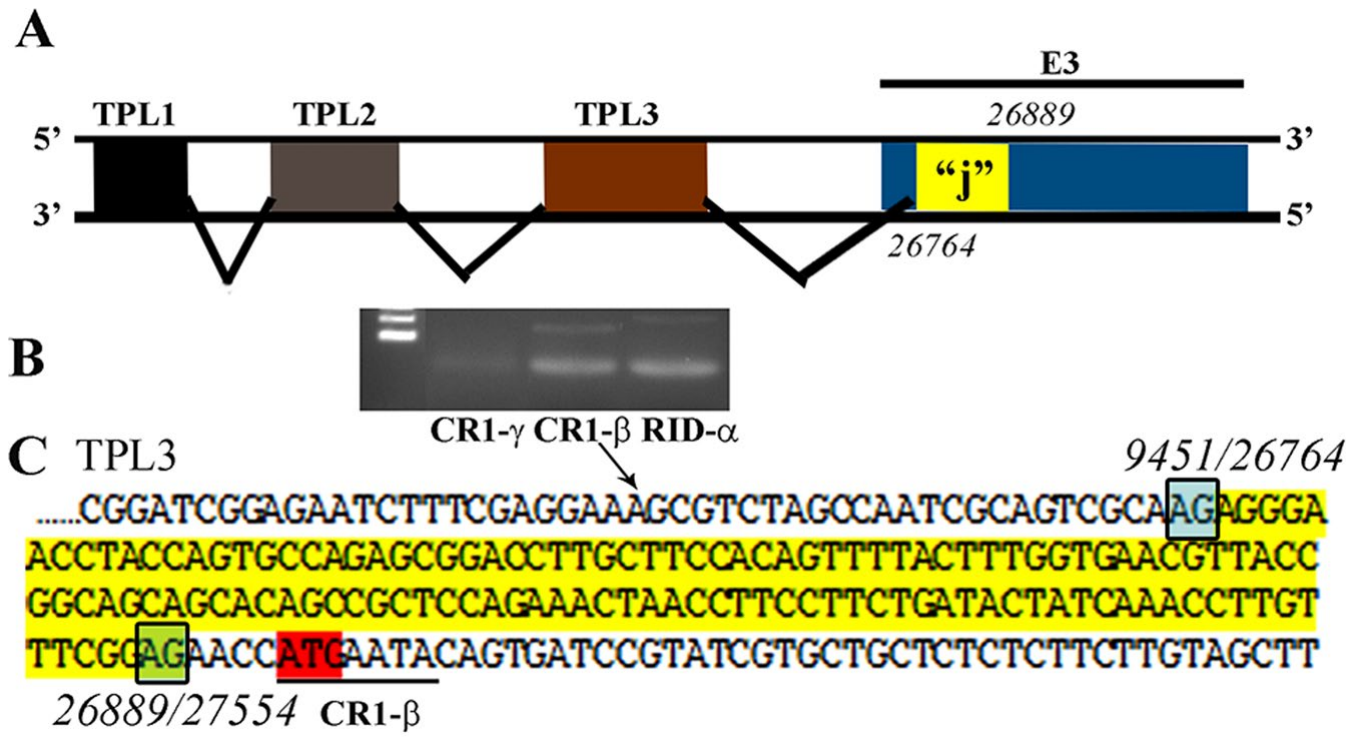
Putative “j”-leader located within the CR1-α E3 gene. (**A**) Schematic for the location of a newly detected leader (“j”-leader) embedded within the E3 CRI-α gene, experimentally determined to be spliced to some, but not all mRNAs of the E3 genes. (**B**) Gel photomicrograph of mRNA transcripts amplified with forward primer from TPL1 and reverse primers from CR1-γ, CR1-β, and RID-α. Primers were chosen to elicit similarly sized bands to facilitate subsequent sequencing. (**C**) Nucleotide sequence of the PCR product for CR1-β. The putative j-leader sequence and splice sites are shown in yellow and green, respectively. Note an additional 4 nucleotide 5′UTR (AACC) prior to the CR1-β start site (red). The 5′UTR in (**C**) prior to the splice site for the j-leader is from TPL3.

To test whether there might be other previously undescribed leaders for mRNAs from the major late promoter transcription unit, using a forward primer from TPL1 and a reverse primers for each E3 and late gene, we performed RT-PCR analysis at 24 hpi and sequenced the PCR products. By this approach, we found a previously undescribed leader sequence at position 26764–26889 (126 nt) in the HAdV-D37 genome (Fig. 5), from within the CR1-α E3 gene. This putative leader sequence, (the “j”-leader), was spliced to mRNAs for six of the E3 genes: gp19 K, CR1-β (shown), CR1-γ, the polycistronic RID-α, and RID-β, and 14.7 K, but not mRNAs for the 12.5 K and the CR1-α genes. The putative j-leader was also found in transcripts for the fiber protein. To investigate whether other HAdV-D types also possess a similar sequence, we blasted the HAdV-D37 j-leader sequence and found close alignments (>90% nucleotide identity) for four of the six other viruses within the CR1-α proteotype containing HAdV-D37^10^, with just two exceptions (Supplemental Table 7). The putative j-leader in HAdV-D56 was 85.9% identical, and in HAdV-D26 was 79.4% identical, respectively, to HAdV-D37 at the nucleotide level. Therefore, for HAdV-D26 in particular, the putative j-leader sequence is less conserved within the proteotype than the remainder of its CR1-α ORF. The putative HAdV-D37 j-leader is 126 nucleotides in length, and in the whole genome is situated within the y-leader (238 nucleotides in length), ending one nucleotide short of the 3′ end of the y-leader. The CR1-α ORF, which contains the entire y-leader (and putative j-leader) is 591 nucleotides in length.

We did not find a j-leader ortholog in other species, but because of overlap in location within the genome, we then directly compared the j-leader sequence of HAdV-D37 with the HAdV-C2 y-leader. The latter is also embedded within the E3 region (located between the 12.K and the CR1- α gene). We found differences in length (188 nucleotides in HAdV-C2 vs. 126 nucleotides in HAdV-D37), and a percent identity between the HAdV-D j-leader and HAdV-C y-leader of only 53.9% by EMBOSS needle (http://www.ebi.ac.uk/Tools/psa/emboss_needle/nucleotide.html). Identity between the HAdV-C2 and HAdV-D37 y-leaders was only 48.4%. These data suggest that the putative j-leader and the previously described y-leader in HAdV-D are both distinct from the previously described y-leader in HAdV-C. However, the putative j-leader in HAdV-D37 falls within the y-leader, ending one nucleotide from the 3′ end of the y-leader. Their predicted splice sites overlap by one nucleotide (AG for j-leader and GG for y-leader).

### Leader arrangements differ in non-human adenoviruses

HAdVs replicate poorly in other animal species^37^, including murine models^37–40^. We and others have demonstrated transcription of HAdV early genes in mouse cells, however, late gene expression was not detected, suggesting a block in translation^38, 39^. As the 5′UTR plays a decisive role in initiation of translation, we determined the leader sequences in mouse adenovirus type 2 (MAV-2) hexon and penton base mRNA after infection of CMT-93 cells, and identified a bipartite leader (Supplemental Fig. 3A), as was previously shown for fowl adenovirus type 10^41^. We then compared TPLs from one representative of each HAdV species with the MAV-2^42^ leader sequences (Supplemental Fig. 3B). The MAV-2 leader sequences are 20 nucleotides longer than the longest TPL sequences (200 nucleotides: HAdV-C and F). By Clustal Omega comparison, we found differences between BPL1-2 and TPL1-3 ranging from 41.24% (MAV-2 vs. HAdV-A12) to 45.88% (MAV-2 vs. HAdV-B3 and -E4) (Table 4). We additionally assessed homology between individual MAV-2 bipartite leaders and those from HAdVs, finding homologies about 43% (Supplemental Table 8). However, the homologies between individual HAdV TPL and MAV BPL were relatively small, ranging from a low of ~30% when comparing TPL3 of HAdV-A12 to MAV-2 BPL1, to a high of 56% when comparing TPL1 of HAdV-F40 to MAV-2 BPL1. We also aligned the entire TPL1-3 and also TPL1-3 including the i-leader, with BPL1-2, but there was low similarity (~40–45%) (Supplemental Table 9), and no specific regions that aligned better than others (data not shown), suggesting a lack of homology between human and mouse adenovirus leader sequences.

**Table 4:** Comparison of tripartite leaders of representative HAdV types across species with bipartite leaders of MAV-2.

Virus species and type	HAdV-A12	HAdV-B3	HAdV-B11	HAdV-C2	HAdV-D37	HAdV-E4	HAdV-F40	HAdV-G52	MAV-2
MAV-2	41.24	45.88	45.36	42.86	41.99	45.88	41.84	41.75	100.00

## Discussion

Gene expression in mammalian cells is regulated by a cascade of events that includes transcription, post-transcriptional processing including pre-mRNA splicing, mature mRNA nuclear export, translation, and post-translational modification^24, 43–45^. Previously published work indicates that 5′UTRs have significant functional consequences for the regulation of mammalian and viral genes; GC content, length, and secondary structure impact mRNA stability, nuclear export, and translation initiation^24, 43^. However, for the most part the underlying mechanisms in these processes remain poorly characterized. Furthermore, recent findings demonstrate that the stressed mammalian cell is able to initiate translation by a broad array of cap-independent mechanisms^46^. Alternative splicing, and with it the HAdV TPL, was discovered in HAdV-C2 almost 40 years ago^13^. However, very little is known about the TPLs in those HAdVs described later. Additionally, the impact of the TPL on viral gene expression regulation and possibly other functions needs further investigation. In this report, we annotated the TPL sequences for all 72 HAdV genotypes, and further investigated the evolutionary relationships among the TPLs of HAdV species, and their GC content and lengths. We focused on HAdV-D37, a significant human pathogen within HAdV-D, the species with the most members. Because by definition, the 5′UTR of any mRNA consists of the nucleotides located upstream of the AUG, we further investigated the nucleotides between TPL3 splice junctions and AUG, and searched for possible new leader sequences. In a few instances, our splice site predictions were inconsistent with previously reported findings^47^, for example in the GenBank annotations of HAdV-F40 and HAdV-D9. As annotation methods correctly predicted the leaders in HAdV-C2, -C5, and in our hands, the leaders of HAdV-D37 and MAV-2, we used the same methodology for further analysis.

Our comprehensive analysis of the TPLs revealed conserved locations within the HAdV genome. We found low TPL variability within HAdV species and high TPL diversity between species, as well as differences in length in TPL3, and variance in GC content. Interestingly, the start site of TPL1 in all HAdVs, in MAV-2, and in fowl adenovirus^41^, is found 26 nucleotides downstream of the last ”A” of the major late promoter TATA box. This aspect and the conserved length of 41 nucleotides for TPL1 in all HAdV species suggests a crucial role in viral protein expression. Furthermore, HAdV genomes tend to be highly conserved within species^25^. The observed ~10% increase in GC content in TPLs, when compared to the average GC genome content in species A, B1, B2, C, D, indicates high conservation of the TPLs in these species. The below average GC content in species F does not exclude conservation, but requires further investigation. Taken together, differences in TPL sequences between HAdV species suggest that the linear nucleotide sequence may be of less importance than other features, for example, the secondary structure of the leader in each mRNA.

Ribosome shunting, a mechanism of cap-independent translation initiation, was previously shown for HAdVs^21, 22^, and also during expression of heat shock protein 70 (Hsp70)^21, 22^. However, Hsp70 is also able to initiate cap-independent translation by an internal ribosome entry site (IRES) located in its 5′UTR^48^. Very recently, it was reported that cellular stress induces increased 5′UTR methylation and facilitates Hsp70 translation in an N^6^-methyladenosine-dependent manner^46, 49, 50^. These findings, together with variable structures of the HAdV leaders, suggest that HAdVs use more than one translation initiation mechanism. Therefore, from the viewpoint of evolution, TPL diversity would be an important mediator of viral fitness.

The i-leader was first described as a 26 nucleotide leader that precedes a 13.6 K protein, but was also described as a more than 400 nucleotide long leader, located between TPL2 and TPL3^30, 32^. In HAdV-D37 we found two splice variants; splice variant 2 (where the i-leader is not spliced to TPL3) corresponds with a previously predicted 16.57 kDa protein^7^. In HAdV-C2, three splice variants and the expression of an associated protein were shown experimentally^30^. Further research is needed to confirm the expression and function of this putative HAdV protein in HAdV-D37.

In mammalian cells, 5′UTRs play an essential role in regulation of gene expression. The average length of 5′UTRs is ~100 to ~220 nucleotides across eukaryotic species. The nucleotide sequences between splice boundary sites and the first ATG show significant diversity in experiments in HAdV-D37 described herein, and in HAdV-C2, as previously reported^51, 52^. Diversity is particularly evident in the E3 region, which contains the coding regions for proteins known to be important to immune evasion by the virus, suggesting that the E3 region requires finely controlled gene expression. This hypothesis is supported by the sequencing of HAdV-D37 mRNA at 24 hpi, where we found a previously undescribed leader sequence (putative j-leader), spliced to mRNAs of the E3 genes gp19 K, CR1-β, CR1-γ, the polycistronic RID-α and RID-β, 14.7 K, and fiber. This leader, found in the genome at position 26764–26889 (126 nt), is embedded within the CR1-α E3 gene. The absence of the leader in some mRNAs for the above genes suggests complex and precisely coordinated splicing. As this putative leader appears in transcripts from the above six E3 genes and the fiber gene, it is unlikely to be a random splicing artifact. Additionally, the HAdV-C y-leader, with a length of 186 nucleotides, is located between the E3 coding genes 12.5 K and CR1-α. Given the very short CR1-α coding sequence in HAdV-C^9^, the newly detected leader within HAdV-D37 might represent a counterpart to the HAdV-C y-leader. However, we did not detect the x and the z-leaders in HAdV-D37 mRNA. Indeed, the x, y, and z-leaders have not been described in any HAdV species except HAdV-C.

In summary, TPL1-3, i-leader, and putative j-leader sequences appear frequently in HAdV 5′UTRs, but with differences in length, GC content, and secondary structure across species, suggesting potential impact on mRNA stability and translation efficiency. Our findings suggest complex post-transcriptional gene expression regulation that diversifies the virus transcriptome, and results in an adapted replication cycle, and a finely regulated proteome. 5′UTRs play a crucial function along the path from genotype to phenotype, and may be a potential target for medical therapy against adenovirus infections.

## Methods

### Cells, viruses, and infection

Human adenovirus species D type 37 (HAdV-D37, GenBank accession number DQ900900.1) was obtained from the American Type Culture Collection (VR-929, ATCC, Manassas, VA) and grown on A549 cells (CCL-185, ATCC). Murine adenovirus 2 (MAV-2, GenBank accession number HM049560.1), a kind gift from Jason Smith (University of Washington, Seattle), was grown on murine CMT93 cells (CCL-223, ATCC). Viruses were purified by cesium chloride gradient as previously described^38^. Purified virus was titered by Tissue Culture Infectious Dose (TCID) assay on A549 and CMT93 cells, respectively, and confirmed free of endotoxin and mycoplasma contamination by standard assays.

A549 cells were infected with HAdV-D37 at a multiplicity of infection (MOI) of 5 in Dulbecco’s modified eagle medium, supplemented with 2% fetal bovine serum (FBS), penicillin G sulfate, and streptomycin and incubated at 37 °C, 5% CO_2_. One hour post infection, cells were washed twice with 1 × PBS, and fresh media was added. Cultures were allowed to incubate at 37 °C for 24 hours post infection (hpi).

### RNA Isolation, PCR amplification, and sequencing

Total RNA was isolated using the Direct-zol RNA MiniPrep Plus kit (Zymo Research, Irvine, CA) according to the manufacturer’s instructions, and RNA was treated with TURBO DNA-free DNase (Ambion, Austin, TX) to remove any remaining genomic DNA. RNA samples were analyzed on a NanoDrop 2000c (Thermo Scientific, Cambridge, MA), and the iScript cDNA synthesis kit (Bio-Rad, Hercules, CA), and 1 µg RNA was used to generate cDNA according to the manufacturer’s protocol. Primers for PCR and sequencing (Supplemental Table 10) were constructed using the OligoAnalyzer Tool from Integrated DNA Technology (IDT, Coralville, IA) and purchased from IDT. The cDNA product (1 µl) was amplified by PCR in a total volume of 25 µl, composed of 12.5 µl of 2 × GoTaq Green Master Mix (Promega), 9.5 µl ddH_2_O, and 1 µl of each primer (10 pmol/µl), under the conditions: 95 °C (5 min), 25 cycles (to avoid signal saturation) at 95 °C (1 min), 60 °C (1 min), 72 °C (1 min), 92 °C (5 min), and 4 °C for hold.

PCR products were analyzed by agarose gel electrophoresis, visualized after ethidium bromide staining using a Kodak Image Station (Kodak, Medfield, MA), and bands of interest were gel purified using the Illustra GFX PCR and Gel Band Purification kit GE Healthcare, Westborough, MA), and sequenced at the Massachusetts Eye and Ear Sequencing Core Facility, Harvard Medical School. Sanger sequencing was performed using a 3730xl DNA Analyzer (Applied Biosystems, Foster City, CA) and sequences were examined for quality values (QV), a per-base estimate of the base caller accuracy, ranging from 1–99, by using the Applied Biosystems DNA Sequencing Analysis Software 5.1. The QVs of high quality sequences typically ranging from 20 to 50, were used in this study. All experiments were performed in triplicate or greater.

### Sequence and phylogenetic analysis, and splice site prediction

HAdV-D37 mRNA sequences were assessed using Standard Nucleotide Blast (http://blast.ncbi.nlm.nih.gov/), and compared to alternative splice site prediction results (http://wangcomputing.com/assp/)^53^. To annotate the region of tripartite leader 1 (TPL1), TPL2, and TPL3, in all known human adenoviruses (HAdV, GenBank numbers in Supplemental Table 1), and for one representative of each species for the i-leader, the appropriate regions were aligned using the ClustalW option within the software Molecular Evolutionary Genetics Analysis (MEGA) 6.06 (www.megasoftware.net), confirmed by splice site prediction (http://wangcomputing.com/assp/)^53^ and compared to the available TPL annotations in GenBank. GC content was calculated in Excel, and the percent identity matrices were generated using Clustal Omega (http://www.ebi.ac.uk/). Phylogenetic analysis was performed using bootstrap-confirmed neighbor-joining trees (1000 replicates) also designed with MEGA 6.06.

### RNA secondary structure prediction

Secondary structures were predicted using the Mfold program (http://unafold.rna.albany.edu/?q=mfold)^29^ with the following parameters: folding temperature: 37 °C; ionic conditions: 1 M NaCl, no divalent ions; maximum interior loop size: 30; maximum asymmetry of an interior loop: 30. The most optimal secondary structures, with the lowest minimum free energy (MEF, in deltaG), of each leader sequence was chosen and presented in Supplemental Fig. 1.

## Electronic supplementary material

Supplemental Figures and Tables
