# Supplementary material for: The 5′UTR in human adenoviruses: leader diversity in late gene expression
Source: Sci Rep. 2017 Apr 4;7:618. doi: 10.1038/s41598-017-00747-y (PMC5429599; doi:10.1038/s41598-017-00747-y)
Supplement: Supplementary file 1 — Supplemental Figures and Tables [file 41598_2017_747_MOESM1_ESM.pdf]

The 5' UTR in human adenoviruses: leader diversity in late gene expression

Mirja Ramke, Jeong Yoon Lee, David W. Dyer, Donald Seto, Jaya Rajaiya, and James Chodosh

Supplementary Information

SUPPLEMENTAL FIGURE LEGENDS

Supplemental Figure 1. RNA secondary structure prediction.

The RNA secondary structures for (A) HAdV-A12, (B) -B3, (C) -B11, (D) -C2, (E) -D37, (F) -E4, (G) -F40, (H) -G52 were predicted using Mfold. The secondary structure with the minimum free energy, i.e., most likely to form in nature, was chosen, and the energy indicated below each structure. dG: final ΔG for a secondary structure is the sum of the free energies assigned to all of the loops and base pair stacks.

Supplemental Figure 2. Complementarity between TPL1-3 and 18S ribosomal sequence.

(A-C) TPL1-3, respectively, for selected representative virus of each HAdV species. Complementary sequence is shown in yellow and splice sites in green.

Supplemental Figure 3. Mouse adenovirus type 2 has a bipartite leader. (A) Schematic for the mouse adenovirus type 2 (MAV-2) leader structure as determined experimentally for MAV-2 hexon and penton base mRNA after infection of CMT-93 cells for 24 hrs. (B) Percent identity for MAV-2 BPL1-2 as compared with HAdV TPL 1-3, and start and end nucleotide locations and length for BPL 1 and 2.

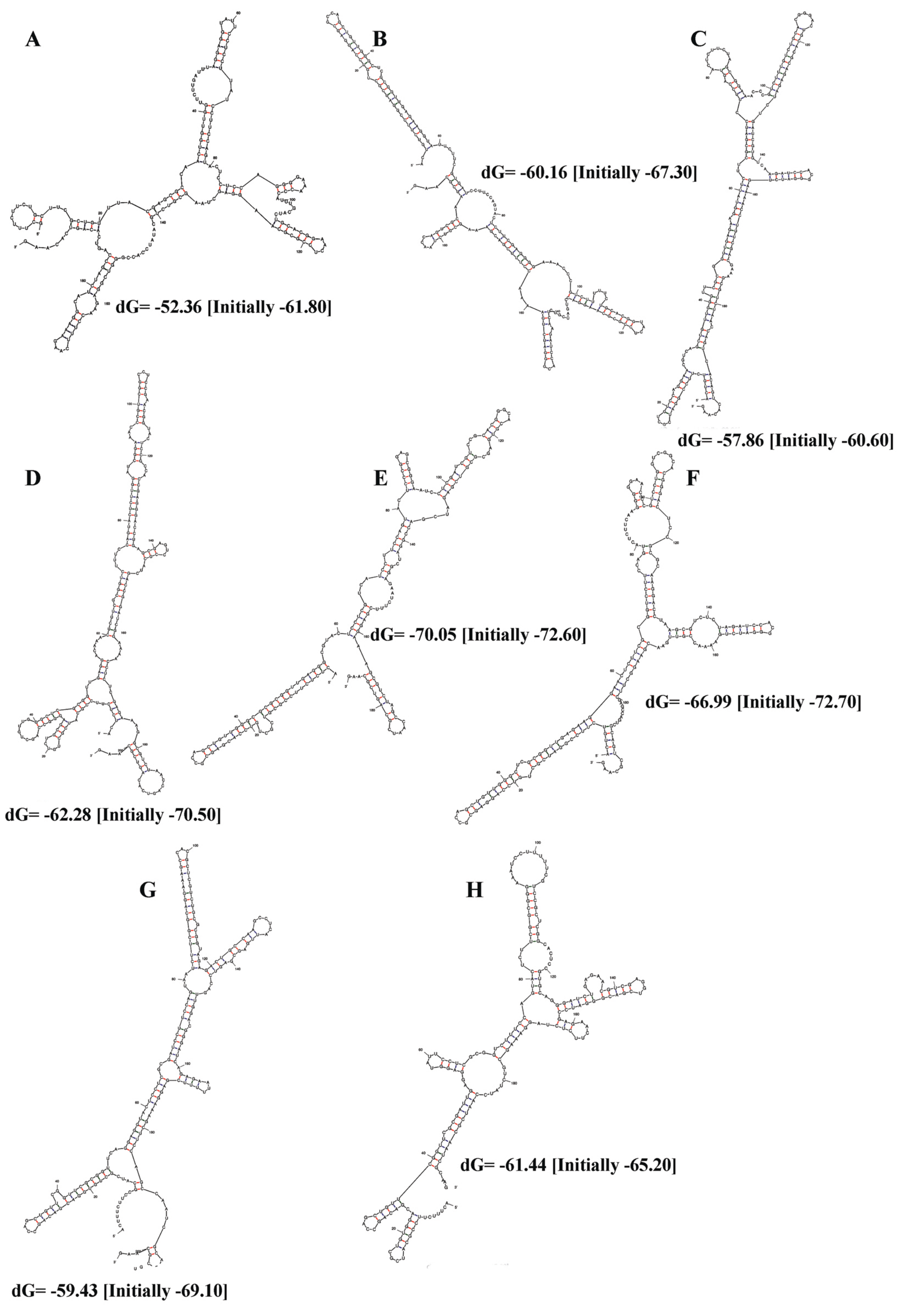
A
dG= -52.36 [Initially -61.80]
B
dG= -60.16 [Initially -67.30]
C
dG= -57.86 [Initially -60.60]
D
dG= -62.28 [Initially -70.50]
E
dG= -70.05 [Initially -72.60]
F
dG= -66.99 [Initially -72.70]
G
dG= -59.43 [Initially -69.10]
H
dG= -61.44 [Initially -65.20]

A.

```
HAdV-A12      GCUUUCUUCUGCUUCGCUGUUUACGAGCGCCAACUGGUUGG
HAdV-B3       ACUGUCUUCCGGAUCGCUGUCCAGGAGCGCCAGCUGUUGGG
HAdV-B11      ACUGUCUUCCGGAUCGCUGUCCAGGAACGUCAGCUGUUGGG
HAdV-C2       ACUCUCUUCCGCAUCGCUGUCUGCGAGGGCCAGCUGUUGGG
HAdV-D37      ACUCUCUUCCGCGUCGCUGUCCACGAGCGCCAGCUGUUGGG
HAdV-E4       ACUGUCUUCCGGAUCGCUGUCCAGGAGCGCCAGCUGUUGGG
HAdV-F40      ACUUUCUUCCGCAUCGCUGUGGACGACAGCCAGCUGUUCGG
HAdV-G52      ACUUUCUUCCGCAUCGCUGUGGACCAGAGCCAGCUGUUCGG
```

B.

```
HAdV-A12      UUCUUGAUUUAGGAGGUAUUCCUCCUUAUCCUUCCAGUACUCCUCCAGGGGAAACCCAUUUGCAUCUGCACG
HAdV-B3       CUCGCGGUUGAGAAGGUAUUCUUCGUGAUCCUUCCAGUACUCUUCGAGGGGAAACCCGUCUUUUUCUGCACG
HAdV-B11      UUCGCGGUUGAGAAGGUAUUCUUCGCGAUCCUUCCAGUACUCUUCUAGCGGAAACCCGUCUUUGUCUGCACG
HAdV-C2       CUCGCGGUUGAGGACAAACUCUUCGCGGUCUUUCCAGUACUCUUGGAUCGGAAACCCGUCGGCCUCCGAACG
HAdV-D37      CGCGCGGUUGAGGGCGUACUCCUCGUCAUCCUUCCAGUACUCCCGGAGCGGGAAUCCUCGAUCGUCCGCACG
HAdV-E4       CUCGCGGUUGAGAAGGAACUCUUCGCGGUCCUUCCAGUACUCUUCAAGGGGGAACCCGUCCUGGUCGGCACG
HAdV-F40      UUCGCGGUUCAGGAGGUACUCCUCGCGAUCCUUCCAGUAAUCUUCGGCAGGAAAGCCACGCUCGUCUGCGCG
HAdV-G52      UUCGCGAUUGAGGAGGUAUUCCUCGCGGUCUUUCCAGUACUCUUCGGCGGGAAAUCCUUUUUCGUCCGCUCG
```

C.

```
HAdV-A12      ---GAACUCCGCGCAAGGAGCUAAGCGUCUGCAUAUCCACCGGGUCGGAGAACCUUUCAAGAAAGGCAUCUAGCCAGUCACAGUCACAAG
HAdV-B3       ---GUACUCCGCGCAAGGACCUGAUCGUCUCAAGAUCCACGGGAUCUGAAAACCUUUCGACGAAAGCGUCUAACCAGUCGCAAUCGCAAG
HAdV-B11      ---GGACUGCACGCAGAGAUCUGAUCGUGUCAAGAUCCACGGGAUCUGAAAACCUUUCGACGAAAGCGUCUAACCAGUCACAGUCACAAG
HAdV-C2       GUACUCCGCCACCGAGGGACCUGAGCGAGUCCGCAUCGACCGGAUCGGAAAACCUCUCGAGAAAGGCGUCUAACCAGUCACAGUCGCAAG
HAdV-D37      ---------------GGCACGUGAGCGCGUCGAGAUCGACCGGAUCGGAGAAUCUUUCGAGGAAAGCGUCUAGCCAAUCGCAGUCGCAAG
HAdV-E4       ---GGACUCCGCGCAAGGACCUAAGCGUCUCUAGAUCCACGGGAUCUGAAAACCGUUGAACGAAGGCUUCGAGCCAGUCGCAGUCGCAAG
HAdV-F40      GUAGAGACUGCCAAGCCUCAUUGAGCGAGUCCAGUUGGACGGGAUCAGAGAAUUUUUCGAGGAAAGCUUCCAGCCAAUCGCAGUCGCAAG
HAdV-G52      ---GCACUCCGUGCAGGGAUCUGAGAACGUCGAGGUCGACGGGAUCCGAGAACUUCUCUAGGAAAGCGUCUAUCCAAUCGCAAUCGCAAG
```

A

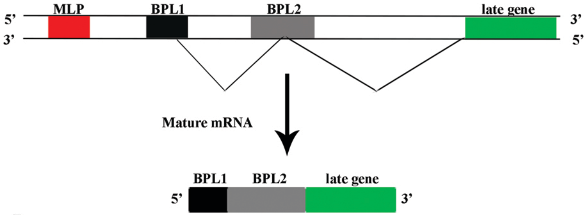

B

Virus species and type	Percent Identity Matrix: BPL1-2							
	HAdV-A12	HAdV-B3	HAdV-B11	HAdV-C2	HAdV-D37	HAdV-E4	HAdV-F40	HAdV-G52
MAV-2	41.24	45.88	45.36	42.86	41.99	45.88	41.84	41.75

Virus species and type	GenBank accession number	BPL1			BPL2			Length BPL1-2
MAV-2	HM049560	Start	End	nt	Start	End	nt	Sum nt
		6251	6332	82	10115	10255	141	223

Supplemental Table 1. Virus genome sequences, GenBank accession numbers, and tripartite leader start and end points and lengths.

Species and type of viruses	GenBank accession number	TPL1			TPL2			TPL3			Length TPL1-3
		**Start**	**End**	**nt**	**Start**	**End**	**nt**	**Start**	**End**	**nt**	**Sum nt**
HAdV-A12	NC_001460	5802	5842	41	6864	6935	72	9370	9456	87	200
HAdV-A18	GU191019.1	5799	5839	41	6861	6932	72	9370	9456	87	200
HAdV-A31	AM749299.1	5715	5755	41	6777	6848	72	9256	9342	87	200
HAdV-A61	JF964962.1	5713	5753	41	6775	6846	72	9254	9340	87	200
HAdV-B3	DQ086466.1	5903	5943	41	6962	7033	72	9477	9563	87	200
HAdV-B7	HQ659699.1	5904	5944	41	6963	7034	72	9478	9564	87	200
HAdV-B11	AF532578.1	5919	5959	41	6978	7049	72	9493	9579	87	200
HAdV-B21	KJ364588.1	5901	5941	41	6960	7031	72	9475	9561	87	200
HAdV-B14	JN032132.1	5939	5979	41	6998	7069	72	9513	9599	87	200
HAdV-B16	JN860680.1	5795	5835	41	6854	6925	72	9369	9455	87	200
HAdV-B34	AY737797.1	5921	5961	41	6981	7052	72	9495	9581	87	200
HAdV-B35	AC_000019.1	5921	5961	41	6980	7051	72	9495	9581	87	200
HAdV-B50	AY737798.1	5900	5940	41	6959	7030	72	9474	9560	87	200
HAdV-B55	FJ643676.1	5925	5965	41	6984	7055	72	9499	9585	87	200
HAdV-B66	JN860676.1	5903	5943	41	6962	7033	72	9477	9563	87	200
HAdV-B68	JN860678.1	5902	5942	41	6961	7032	72	9476	9562	87	200
HAdV-C1	JX173086.1	6062	6102	41	7125	7196	72	9654	9743	90	203
HAdV-C2	J01917.1	6039	6079	41	7101	7172	72	9634	9723	90	203
HAdV-C5	AC_000008.1	6049	6089	41	7111	7182	72	9644	9733	90	203
HAdV-C6	KF268129.1	6064	6104	41	7127	7198	72	9659	9748	90	203
HAdV-C57	HQ003817.1	6057	6097	41	7119	7190	72	9652	9741	90	203
HAdV-D8	AB448769.1	5855	5895	41	6917	6988	72	9369	9443	75	188
HAdV-D9	AJ854486.1	5854	5894	41	6916	6987	72	9374*	9448	75	188
HAdV-D10	JN226746.1	5858	5898	41	6920	6991	72	9378	9452	75	188
HAdV-D13	JN226747.1	5866	5906	41	6928	6999	72	9377	9451	75	188

HAdV-D15	AB562586.1	5862	5902	41	6924	6995	72	9382	9456	75	188
HAdV-D17	HQ910407.1	5846	5886	41	6908	6979	72	9363	9437	75	188
HAdV-D19	JQ326209.1	5837	5877	41	6899	6970	72	9360	9434	75	188
HAdV-D20	JN226749.1	5844	5884	41	6906	6977	72	9364	9438	75	188
HAdV-D22	FJ619037.1	5847	5887	41	6909	6980	72	9367	9441	75	188
HAdV-D23	JN226750.1	5846	5886	41	6908	6979	72	9363	9437	75	188
HAdV-D24	JN226751.1	5851	5891	41	6913	6984	72	9371	9445	75	188
HAdV-D25	JN226752.1	5848	5888	41	6910	6981	72	9368	9442	75	188
HAdV-D26	EF153474.1	5853	5893	41	6915	6986	72	9373	9447	75	188
HAdV-D27	JN226753.1	5858	5898	41	6920	6991	72	9378	9452	75	188
HAdV-D28	FJ824826.1	5849	5889	41	6911	6982	72	9369	9443	75	188
HAdV-D29	JN226754.1	5858	5898	41	6920	6991	72	9378	9452	75	188
HAdV-D30	JN226755.1	5852	5892	41	6914	6985	72	9372	9446	75	188
HAdV-D32	JN226756.1	5852	5892	41	6914	6985	72	9372	9446	75	188
HAdV-D33	JN226758.1	5853	5893	41	6915	6986	72	9373	9447	75	188
HAdV-D36	GQ384080.1	5848	5888	41	6910	6981	72	9368	9442	75	188
HAdV-D37	DQ900900.1	5857	5897	41	6919	6990	72	9377	9451	75	188
HAdV-D38	JN226759.1	5864	5904	41	6926	6997	72	9387	9461	75	188
HAdV-D39	JN226760.1	5855	5895	41	6917	6988	72	9375	9449	75	188
HAdV-D42	JN226761.1	5849	5889	41	6911	6982	72	9372	9446	75	188
HAdV-D43	JN226762.1	5852	5892	41	6914	6985	72	9375	9449	75	188
HAdV-D44	JN226763.1	5854	5894	41	6916	6987	72	9374	9448	75	188
HAdV-D45	JN226764.1	5849	5889	41	6911	6982	72	9366	9440	75	188
HAdV-D46	AY875648.1	5850	5890	41	6912	6983	72	9367	9441	75	188
HAdV-D47	JN226757.1	5853	5893	41	6915	6986	72	9373	9447	75	188
HAdV-D48	EF153473.1	5850	5890	41	6912	6983	72	9370	9444	75	188
HAdV-D49	DQ393829.1	5849	5889	41	6911	6982	72	9369	9443	75	188
HAdV-D51	JN226765.1	5848	5888	41	6910	6981	72	9365	9439	75	188
HAdV-D53	AB605246.1	5858	5898	41	6920	6991	72	9378	9452	75	188
HAdV-D54	NC_012959.1	5860	5900	41	6922	6993	72	9374	9448	75	188
HAdV-D56	HM770721.2	5857	5897	41	6919	6990	72	9377	9451	75	188
HAdV-D58	HQ883276.1	5859	5899	41	6921	6992	72	9382	9456	75	188
HAdV-D59	JF799911.1	5856	5896	41	6918	6989	72	9376	9450	75	188
HAdV-D60	HQ007053.1	5852	5892	41	6914	6985	72	9372	9446	75	188

HAdV-D62	JN162671.1	5852	5892	41	6914	6985	72	9366	9440	75	188
HAdV-D63	JN935766.1	5852	5892	41	6914	6985	72	9372	9446	75	188
HAdV-D64	EF121005.1	5858	5898	41	6920	6991	72	9378	9452	75	188
HAdV-D65	AP012285.1	5850	5890	41	6912	6983	72	9370	9444	75	188
HAdV-D67	AP012302.1	5851	5891	41	6913	6984	72	9371	9445	75	188
HAdV-D69	JN226748.1	5846	5886	41	6908	6979	72	9366	9440	75	188
HAdV-D70	KP641339.1	5858	5898	41	6920	6991	72	9381	9455	75	188
HAdV-D71	KF268207.1	5848	5888	41	6910	6981	72	9368	9442	75	188
HAdV-D72	KF268335.1	5847	5887	41	6909	6980	72	9364	9438	75	188
HAdV-E4	NC_003266.2	5885	5925	41	6947	7018	72	9456	9542	87	200
HAdV-F40**	NC_001454.1	5599	5639	41	6661	6732	72	9167	9256	90	203
HAdV-F41	AB728839.1	5590	5630	41	6652	6723	72	9161	9250	90	203
HAdV-G52	DQ923122.2	5548	5588	41	6610	6681	72	9110	9196	87	200
**Murine**											
MAV-2	HM049560.1	6251	6332	82	10115	10255	141				223

Only HAdV-D37 annotations were determined by direct sequencing of mRNA.

*In GenBank, HAdV-D9 TPL3 begins at nucleotide no. 9271.

**In GenBank, HAdV-F40 TPL 1 and 3 are not annotated.

Supplemental Table 2. Identity comparisons for TPL1,2, and 3 for representative viruses from each HAdV species.

species and type	Percent Identity Matrix: TPL 1							
	**HAdV-A12**	**HAdV-B3**	**HAdV-B11**	**HAdV-C2**	**HAdV-D37**	**HAdV-E4**	**HAdV-F40**	**HAdV-G52**
**HAdV-A12**	100.00							
**HAdV-B3**	73.17	100.00						
**HAdV-B11**	68.29	95.12	100.00					
**HAdV-C2**	75.61	85.37	80.49	100.00				
**HAdV-D37**	78.05	90.24	85.37	90.24	100.00			
**HAdV-E4**	73.17	100.00	95.12	85.37	90.24	100.00		
**HAdV-F40**	75.61	80.49	78.05	82.93	82.93	80.49	100.00	
**HAdV-G52**	75.61	80.49	75.61	82.93	82.93	80.49	95.12	100.00

species and type	Percent Identity Matrix: TPL 2							
	**HAdV-A12**	**HAdV-B3**	**HAdV-B11**	**HAdV-C2**	**HAdV-D37**	**HAdV-E4**	**HAdV-F40**	**HAdV-G52**
**HAdV-A12**	100.00							
**HAdV-B3**	77.78	100.00						
**HAdV-B11**	76.39	93.06	100.00					
**HAdV-C2**	63.89	76.39	76.39	100.00				
**HAdV-D37**	66.67	72.22	70.83	70.83	100.00			
**HAdV-E4**	68.06	86.11	86.11	79.17	70.83	100.00		
**HAdV-F40**	70.83	75.00	77.78	68.06	72.22	73.61	100.00	
**HAdV-G52**	75.00	77.78	79.17	73.61	70.83	75.00	80.56	100.00

species and type	Percent Identity Matrix: TPL 3							
	**HAdV-A12**	**HAdV-B3**	**HAdV-B11**	**HAdV-C2**	**HAdV-D37**	**HAdV-E4**	**HAdV-F40**	**HAdV-G52**
**HAdV-A12**	100.00							
**HAdV-B3**	77.01	100.00						
**HAdV-B11**	74.71	88.51	100.00					
**HAdV-C2**	73.56	74.71	74.71	100.00				
**HAdV-D37**	73.33	78.67	77.33	81.33	100.00			
**HAdV-E4**	80.46	86.21	79.31	68.97	73.33	100.00		
**HAdV-F40**	58.62	62.07	62.07	62.22	80.00	60.92	100.00	
**HAdV-G52**	67.82	77.01	73.56	68.97	81.33	70.11	64.37	100.00

Supplemental Table 3. Sequence and GC content for TPL1, by virus.

Type no.	TPL1	A	T	G	C	Sum	A/T	G/C
HAdV-A12	GCTTTCTTCTGCTTCGCTGTTTACGAGCGCCAACTGGTTGG	4	15	11	11	41	46.34	53.66
HAdV-A18	GCTTTCTTCTGCATCGCTGTTTACGAGCGCCAGCTGTTGGG	4	14	12	11	41	43.90	56.10
HAdV-A31	GCTGTCTTCGGCTTCGCTGTTTACGAGCGCCAGCTGTTGCG	3	13	13	12	41	39.02	60.98
HAdV-A61	GCTGTCTTCGGCTTCGCTGTTTACGAGCGCCAGCTGTTGGG	3	13	14	11	41	39.02	60.98
HAdV-B3	ACTGTCTTCCGGATCGCTGTCCAGGAGCGCCAGCTGTTGGG	5	10	14	12	41	36.59	63.41
HAdV-B7	ACTGTCTTCCGGATCGCTGTCCAGGAGCGCCAGCTGTTGGG	5	10	14	12	41	36.59	63.41
HAdV-B11	ACTGTCTTCCGGATCGCTGTCCAGGAACGTCAGCTGTTGGG	6	11	13	11	41	41.46	58.54
HAdV-B14	ACTGTCTTCCGGATCGCTGTCCAGGAACGTCAGCTGTTGGG	6	11	13	11	41	41.46	58.54
HAdV-B16	ACTGTCTTCCGGATCGCTGTCCAGGAGCGCCAGCTGTTGGG	5	10	14	12	41	36.59	63.41
HAdV-B21	ACTGTCTTCAGGATCGCTGTCCAGGAGCGCCAGCTGTTGGG	6	10	14	11	41	39.02	60.98
HAdV-B34	ACTGTCTTCCGGATCGCTGTCCAGGAACGTCAGCTGTTGGG	6	11	13	11	41	41.46	58.54
HAdV-B35	ACTGTCTTCCGGATCGCTGTCCAGGAACGTCAGCTGTTGGG	6	11	13	11	41	41.46	58.54
HAdV-B50	ACTGTCTTCCGGATCACTGTCCAGGAGCGCCAGCTGTTGGG	6	10	13	12	41	39.02	60.98
HAdV-B55	ACTGTCTTCCGGATCGCTGTCCAGGAACGTCAGCTGTTGGG	6	11	13	11	41	41.46	58.54
HAdV-B66	ACTGTCTTCCGGATCGCTGTCCAGGAGCGCCAGCTGTTGGG	5	10	14	12	41	36.59	63.41
HAdV-B68	ACTGTCTTCAGGATCGCTGTCCAGGAGCGCCAGCTGTTGGG	6	10	14	11	41	39.02	60.98
HAdV-C1	ACTCTCTTCCGCATCGCTGTCTGCGAGGGCCAGCTGTTGGG	4	11	13	13	41	36.59	63.41
HAdV-C2	ACTCTCTTCCGCATCGCTGTCTGCGAGGGCCAGCTGTTGGG	4	11	13	13	41	36.59	63.41
HAdV-C5	ACTCTCTTCCGCATCGCTGTCTGCGAGGGCCAGCTGTTGGG	4	11	13	13	41	36.59	63.41
HAdV-C6	ACTCTCTTCCGCATCGCTGTCTGCGAGGGCCAGCTGTTGGG	4	11	13	13	41	36.59	63.41
HAdV-C57	ACTCTCTTCCGCATCGCTGTCTGCGAGGGCCAGCTGTTGGG	4	11	13	13	41	36.59	63.41
HAdV-D8	ACTCTCTTCCGCGTCGCTGTCCACGAGCGTCAGCTGTTGGG	4	11	12	14	41	36.59	63.41
HAdV-D9	ACTCTCTTCCGCGTCGCTGTCCACGAGCGCCAGCTGTTGGG	4	10	12	15	41	34.15	65.85
HAdV-D10	ACTCTCTTCCGCGTCGCTGTCCACGAGCGCCAGCTGTTGGG	4	10	12	15	41	34.15	65.85
HAdV-D13	ACTCTCTTCCGCGTCGCTGTCCACGAGCGCCAGCTGTTGGG	4	10	12	15	41	34.15	65.85
HAdV-D15	ACTCTCTTCCGCGTCGCTGTCCACGAGCGCCAGCTGTTGGG	4	10	12	15	41	34.15	65.85
HAdV-D17	ACTCTCTTCCGCGTCGCTGTCCACGAGCGCCAGCTGTTGGG	4	10	12	15	41	34.15	65.85
HAdV-D19	ACTCTCTTCCGCGTCGCTGTCCACGAGCGCCAGCTGTTGGG	4	10	12	15	41	34.15	65.85
HAdV-D20	ACTCTCTTCCGCGTCGCTGTCCACGAGCGCCAGCTGTTGGG	4	10	12	15	41	34.15	65.85

HAdV-D22	ACTCTCTTCCGCGTCGCTGTCCACGAGCGCCAGCTGTTGGG	4	10	12	15	41	34.15	65.85
HAdV-D23	ACTCTCTTCCGCGTCGCTGTCCACGAGCGCCAGCTGTTGGG	4	10	12	15	41	34.15	65.85
HAdV-D24	ACTCTCTTCCGCGTCGCTGTCCACGAGCGCCAGCTGTTGGG	4	10	12	15	41	34.15	65.85
HAdV-D25	ACTCTCTTCCGCGTCGCTGTCCACGAGCGCCAGCTGTTGGG	4	10	12	15	41	34.15	65.85
HAdV-D26	ACTCTCTTCCGCGTCGCTGTCCACGAGCGCCAGCTGTTGGG	4	10	12	15	41	34.15	65.85
HAdV-D27	ACTCTCTTCCGCGTCGCTGTCCACGAGCGCCAGCTGTTGGG	4	10	12	15	41	34.15	65.85
HAdV-D28	ACTCTCTTCCGCGTCGCTGTCCACGAGCGCCAGCTGTTGGG	4	10	12	15	41	34.15	65.85
HAdV-D29	ACTCTCTTCCGCGTCGCTGTCCACGAGCGCCAGCTGTTGGG	4	10	12	15	41	34.15	65.85
HAdV-D30	ACTCTCTTCCGCGTCGCTGTCCACGAGCGCCAGCTGTTGGG	4	10	12	15	41	34.15	65.85
HAdV-D32	ACTCTCTTCCGCGTCGCTGTCCACGAGCGCCAGCTGTTGGG	4	10	12	15	41	34.15	65.85
HAdV-D33	ACTCTCTTCCGCGTCGCTGTCCACGAGCGCCAGCTGTTGGG	4	10	12	15	41	34.15	65.85
HAdV-D36	ACTCTCTTCCGCGTCGCTGTCCACGAGCGCCAGCTGTTGGG	4	10	12	15	41	34.15	65.85
HAdV-D37	ACTCTCTTCCGCGTCGCTGTCCACGAGCGCCAGCTGTTGGG	4	10	12	15	41	34.15	65.85
HAdV-D38	ACTCTCTTCCGCGTCGCTGTCCACGAGCGCCAGCTGTTGGG	4	10	12	15	41	34.15	65.85
HAdV-D39	ACTCTCTTCCGCGTCGCTGTCCACGAGCGCCAGCTGTTGGG	4	10	12	15	41	34.15	65.85
HAdV-D42	ACTCTCTTCCGCGTCGCTGTCCACGAGCGCCAGCTGTTGGG	4	10	12	15	41	34.15	65.85
HAdV-D43	ACTCTCTTCCGCGTCGCTGTCCACGAGCGCCAGCTGTTGGG	4	10	12	15	41	34.15	65.85
HAdV-D44	ACTCTCTTCCGCGTCGCTGTCCACGAGCGCCAGCTGTTGGG	4	10	12	15	41	34.15	65.85
HAdV-D45	ACTCTCTTCCGCGTCGCTGTCCACGAGCGCCAGCTGTTGGG	4	10	12	15	41	34.15	65.85
HAdV-D46	ACTCTCTTCCGCGTCGCTGTCCACGAGCGCCAGCTGTTGGG	4	10	12	15	41	34.15	65.85
HAdV-D47	ACTCTCTTCCGCGTCGCTGTCCACGAGCGCCAGCTGTTGGG	4	10	12	15	41	34.15	65.85
HAdV-D48	ACTCTCTTCCGCGTCGCTGTCCACGAGCGCCAGCTGTTGGG	4	10	12	15	41	34.15	65.85
HAdV-D49	ACTCTCTTCCGCGTCGCTGTCCACGAGCGCCAGCTGTTGGG	4	10	12	15	41	34.15	65.85
HAdV-D51	ACTCTCTTCCGCGTCGCTGTCCACGAGCGCCAGCTGTTGGG	4	10	12	15	41	34.15	65.85
HAdV-D53	ACTCTCTTCCGCGTCGCTGTCCACGAGCGCCAGCTGTTGGG	4	10	12	15	41	34.15	65.85
HAdV-D54	ACTCTCTTCCGCGTCGCTGTCCACGAGCGCCAGCTGTTGGG	4	10	12	15	41	34.15	65.85
HAdV-D56	ACTCTCTTCCGCGTCGCTGTCCACGAGCGCCAGCTGTTGGG	4	10	12	15	41	34.15	65.85
HAdV-D58	ACTCTCTTCCGCGTCGCTGTCCACGAGCGCCAGCTGTTGGG	4	10	12	15	41	34.15	65.85
HAdV-D59	ACTCTCTTCCGCGTCGCTGTCCACGAGCGCCAGCTGTTGGG	4	10	12	15	41	34.15	65.85
HAdV-D60	ACTCTCTTCCGCGTCGCTGTCCACGAGCGCCAGCTGTTGGG	4	10	12	15	41	34.15	65.85
HAdV-D62	ACTCTCTTCCGCGTCGCTGTCCACGAGCGCCAGCTGTTGGG	4	10	12	15	41	34.15	65.85
HAdV-D63	ACTCTCTTCCGCGTCGCTGTCCACGAGCGCCAGCTGTTGGG	4	10	12	15	41	34.15	65.85
HAdV-D64	ACTCTCTTCCGCGTCGCTGTCCACGAGCGCCAGCTGTTGGG	4	10	12	15	41	34.15	65.85
HAdV-D65	ACTCTCTTCCGCGTCGCTGTCCACGAGCGCCAGCTGTTGGG	4	10	12	15	41	34.15	65.85

HAdV-D67	ACTCTCTTCCGCGTCGCTGTCCACGAGCGCCAGCTGTTGGG	4	10	12	15	41	34.15	65.85
HAdV-D69	ACTCTCTTCCGCGTCGCTGTCTACGAGCGCCAGCTGTTGGG	4	11	12	14	41	36.59	63.41
HAdV-D70	ACTCTCTTCCGCGTCGCTGTCCACGAGCGCCAGCTGTTGGG	4	10	12	15	41	34.15	65.85
HAdV-D71	ACTCTCTTCCGCGTCGCTGTCCACGAGCGCCAGCTGTTGGG	4	10	12	15	41	34.15	65.85
HAdV-D72	ACTCTCTTCCGCGTCGCTGTCCACGAGCGCCAGCTGTTGGG	4	10	12	15	41	34.15	65.85
HAdV-E4	ACTGTCTTCCGGATCGCTGTCCAGGAGCGCCAGCTGTTGGG	5	10	14	12	41	36.59	63.41
HAdV-F40	ACTTTCTTCCGCATCGCTGTGGACGACAGCCAGCTGTTCGG	6	11	11	13	41	41.46	58.54
HAdV-F41	ACTTTCTTCCGCATCGCTGTGGGCAAGAGCCAGCTGTTCGG	6	11	12	12	41	41.46	58.54
HAdV-G52	ACTTTCTTCCGCATCGCTGTGGACCAGAGCCAGCTGTTCGG	6	11	11	13	41	41.46	58.54

Nucleotide differences from the paradigm (first) type in each species shown in red.

Supplemental Table 4. Sequence and GC content for TPL2, by virus.

Type no.	TPL2	A	T	G	C	Sum	A/T	G/C
HAdV-A12	TTCTTGATTTAGGAGGTATTCCTCCTTATCCTTCCAGTACTCCTCCAGGGGAAACCCATTTGCATCTGCACG	14	24	13	21	72	52.78	47.22
HAdV-A18	TTGTTGGTTTAGGAGGTATTCTTCCTTGTCCTTCCAATACTCTTCCACGGGAAAACCGTCTGCATTTGCACG	13	26	15	18	72	54.17	45.83
HAdV-A31	TTCTTGGTTTAGGAGGTATTCCTGTTTATCCTTCCAATACTCCTCTACAGGGAAACCATCTGCATCAGCGCG	15	24	14	19	72	54.17	45.83
HAdV-A61	TTCTTGGTTTAGGAGGTATTCCTGTTTATCCTTCCAATACTCCTCTAAAGGGAAACCATCTGCATCAGCGCG	16	24	14	18	72	55.56	44.44
HAdV-B3	CTCGCGGTTGAGAAGGTATTCTTCGTGATCCTTCCAGTACTCTTCGAGGGGAAACCCGTCTTTTTCTGCACG	12	23	18	19	72	48.61	51.39
HAdV-B7	CTCGCGGTTGAGAAGGTATTCTTCGTGATCCTTCCAGTACTCTTCGAGGGGAAACCCGTCTTTTTCTGCACG	12	23	18	19	72	48.61	51.39
HAdV-B11	TTCGCGGTTGAGAAGGTATTCTTCGCGATCCTTCCAGTACTCTTCTAGCGGAAACCCGTCTTTGTCTGCACG	12	23	17	20	72	48.61	51.39
HAdV-B21	CTCGCGGTTGAGAAGGTATTCTTCGCGATCCTTCCAGTACTCTTCGAGGGGAAACCCGTCTTTTTCTGCACG	12	22	18	20	72	47.22	52.78
HAdV-B14	TTCGCGGTTGAGAAGGTATTCTTCGCGATCCTTCCAGTACTCTTCTAGCGGAAACCCGTCTTTGTCTGCACG	12	23	17	20	72	48.61	51.39
HAdV-B16	CTCGCGGTTGAGAAGGTATTCTTCGCGATCCTTCCAGTACTCTTCGAGGGGAAACCCGTCTTTTTCTGCACG	12	22	18	20	72	47.22	52.78
HAdV-B34	TTCGCGGTTGAGAAGGTATTCTTCGCGATCCTTCCAGTACTCTTCTAGCGGAAACCCGTCTTTGTCTGCACG	12	23	17	20	72	48.61	51.39
HAdV-B35	TTCGCGGTTGAGAAGGTATTCTTCGCGATCCTTCCAGTACTCTTCTAGCGGAAACCCGTCTTTGTCTGCACG	12	23	17	20	72	48.61	51.39
HAdV-B50	CTCGCGGTTGAGAAGGTATTCTTCGCGATCCTTCCAGTACTCTTCGAGGGGAAACCCGTCTTTTTCTGCACG	12	22	18	20	72	47.22	52.78
HAdV-B55	TTCGCGGTTCAGAAGGTATTCTTCGCGATCCTTCCAGTACTCTTCTAGCGGAAACCCGTCTTTGTCTGCACG	12	23	16	21	72	48.61	51.39
HAdV-B66	CTCGCGGTTGAGAAGGTATTCTTCGCGATCCTTCCAGTACTCTTCGAGGGGAAACCCGTCTTTTTCTGCACG	12	22	18	20	72	47.22	52.78
HAdV-B68	CTCGCGGTTGAGAAGGTATTCTTCGCGATCCTTCCAGTACTCTTCGAGGGGAAACCCGTCTTTTTCTGCACG	12	22	18	20	72	47.22	52.78
HAdV-C1	CTCGCGGTTGAGGACAAACTCTTCGCGGTCTTTCCAGTACTCTTGGATCGGAAACCCGTCGGCCTCCGAACG	13	17	19	23	72	41.67	58.33
HAdV-C2	CTCGCGGTTGAGGACAAACTCTTCGCGGTCTTTCCAGTACTCTTGGATCGGAAACCCGTCGGCCTCCGAACG	13	17	19	23	72	41.67	58.33
HAdV-C5	CTCGCGGTTGAGGACAAACTCTTCGCGGTCTTTCCAGTACTCTTGGATCGGAAACCCGTCGGCCTCCGAACG	13	17	19	23	72	41.67	58.33
HAdV-C6	CTCGCGGTTGAGGACAAACTCTTCGCGGTCTTTCCAGTACTCTTGGATCGGAAACCCGTCGGCCTCCGAACG	13	17	19	23	72	41.67	58.33
HAdV-C57	CTCGCGGTTGAGGACAAACTCTTCGCGGTCTTTCCAGTACTCTTGGATCGGAAACCCGTCGGCCTCCGAACG	13	17	19	23	72	41.67	58.33
HAdV-D8	CTCGCGGTTAAGGGCGTACTCCTCGTCATCCTTCCAGTACTCCCGGAGCGGGAATCCTTGATCGTCCGCACG	11	17	19	25	72	38.89	61.11
HAdV-D9	CTCGCGGTTGAGGGCGTACTCCTCGTCATCCTTCCAGTACTCCCGGAGCGGGAATCCTCGATCGTCCGCACG	10	16	20	26	72	36.11	63.89
HAdV-D10	CTCGCGGTTGAGGGCGTACTCCTCGTCATCCTTCCAGTACTCCCGGAGCGGGAATCCTCGATCGTCCGCACG	10	16	20	26	72	36.11	63.89
HAdV-D13	CTCGCGGTTGAGGGCGTACTCCTCGTCATCCTTCCAGTACTCCCGGAGCGGGAATCCTCGATCGTCCGCACG	10	16	20	26	72	36.11	63.89
HAdV-D15	CTCGCGGTTGAGGGCATACTCCTCGTCATCCTTCCAGTACTCCCGGAGCGGGAATCCTCGATCGTCCGCACG	11	16	19	26	72	37.50	62.50
HAdV-D17	CTCGCGGTTGAGGGCGTATTCCTCGTCATCCTTCCAGTACTCCCGGAGCGGGAATCCTCGATCGTCCGCACG	10	17	20	25	72	37.50	62.50
HAdV-D19	CTCGCGGTTGAGGGCGTATTCCTCGTCATCCTTCCAGTACTCCCGGAGCGGGAATCCTAGATCGTCCGCACG	11	17	20	24	72	38.89	61.11
HAdV-D20	CTCGCGGTTGAGGGCGTACTCCTCGTCATCCTTCCAGTACTCCCGGAGCGGGAATCCTCGATCGTCCGCACG	10	16	20	26	72	36.11	63.89

HAdV-D22	CTCGCGGTTGAGGGCGTACTCCTCGTCATCCTTCCAGTACTCCCGGAGCGGGAATCCTCGATCGTCCGCACG	10	16	20	26	72	36.11	63.89
HAdV-D23	CTCGCGGTTGAGGGCGTACTCCTCGTCATCCTTCCAATACTCCCGGAGCGGGAATCCTCGATCGTCCGCACG	11	16	19	26	72	37.50	62.50
HAdV-D24	CTCGCGGTTGAGGGCGTACTCCTCGTCATCCTTCCAGTACTCCCGGAGCGGGAATCCTCGATCGTCCGCACG	10	16	20	26	72	36.11	63.89
HAdV-D25	CTCGCGGTTGAGGGCGTATTCCTCGTCATCCTTCCAGTACTCCCGGAGCGGGAATCCTCGATCGTCCGCACG	10	17	20	25	72	37.50	62.50
HAdV-D26	CTCGCGGTTGAGGGCGTATTCCTCGTCATCCTTCCAGTACTCCCGGAGCGGGAATCCTCGATCGTCCGCACG	10	17	20	25	72	37.50	62.50
HAdV-D27	CTCGCGGTTGAGGGCGTATTCCTCGTCATCCTTCCAGTACTCCCGGAGCGGGAATCCTCGATCGTCCGCACG	10	17	20	25	72	37.50	62.50
HAdV-D28	CTCGCGGTTGAGGGCGTACTCCTCGTCATCCTTCCAGTACTCCCGGAGCGGGAATCCTCGATCGTCCGCACG	10	16	20	26	72	36.11	63.89
HAdV-D29	CTCGCGGTTGAGGGCGTACTCCTCGTCATCCTTCCAGTACTCCCGGAGCGGGAATCCTCGATCGTCCGCACG	10	16	20	26	72	36.11	63.89
HAdV-D30	CTCGCGGTTGAGGGCGTACTCCTCGTCATCCTTCCAGTACTCCCGGAGCGGGAATCCTCGATCGTCCGCACG	10	16	20	26	72	36.11	63.89
HAdV-D32	CTCGCGGTTGAGGGCGTACTCCTCGTCATCCTTCCAATACTCCCGGAGCGGGAATCCTCGATCGTCCGCACG	11	16	19	26	72	37.50	62.50
HAdV-D33	CTCGCGGTTGAGGGCATACTCCTCGTCATCCTTCCAGTACTCCCGGAGCGGGAATCCTCGATCGTCCGCACG	11	16	19	26	72	37.50	62.50
HAdV-D36	CTCGCGGTTGAGGGCGTACTCCTCGTCATCCTTCCAGTACTCCCGGAGCGGGAATCCTCGATCGTCCGCACG	10	16	20	26	72	36.11	63.89
HAdV-D37	CGCGCGGTTGAGGGCGTACTCCTCGTCATCCTTCCAGTACTCCCGGAGCGGGAATCCTCGATCGTCCGCACG	10	15	21	26	72	34.72	65.28
HAdV-D38	CTCGCGGTTGAGGGCGTACTCCTCGTCATCCTTCCAATACTCCCGGAGCGGGAATCCTCGATCGTCCGCACG	11	16	19	26	72	37.50	62.50
HAdV-D39	CTCGCGGTTGAGGGCGTACTCCTCGTCATCCTTCCAGTACTCCCGGAGCGGGAATCCTCGATCGTCCGCACG	10	16	20	26	72	36.11	63.89
HAdV-D42	CTCGCGGTTGAGGGCGTACTCCTCGTCATCCTTCCAGTACTCCCGGAGCGGGAATCCTCGATCGTCCGCACG	10	16	20	26	72	36.11	63.89
HAdV-D43	CTCGCGGTTGAGGGCGTACTCCTCGTCATCCTTCCAGTACTCCCGGAGCGGGAATCCTCGATCGTCCGCACG	10	16	20	26	72	36.11	63.89
HAdV-D44	CTCGCGGTTGAGGGCATACTCCTCGTCATCCTTCCAGTACTCCCGGAGCGGGAATCCTCGATCGTCCGCACG	11	16	19	26	72	37.50	62.50
HAdV-D45	CTCGTGGTTGAGGGCATACTCCTCGTCATCCTTCCAGTACTCCCGGAGCGGGAATCCTCGATCGTCCGCACG	11	17	19	25	72	38.89	61.11
HAdV-D46	CTCGCGGTTGAGGGCATACTCCTCGTCATCCTTCCAGTACTCCCGGAGCGGGAATCCTCGATCGTCCGCACG	11	16	19	26	72	37.50	62.50
HAdV-D47	CTCGCGGTTGAGGGCGTACTCCTCGTCATCCTTCCAGTACTCCCGGAGCGGGAATCCTCGATCGTCCGCACG	10	16	20	26	72	36.11	63.89
HAdV-D48	CTCGCGGTTGAGGGCATACTCCTCGTCATCCTTCCAGTACTCCCGGAGCGGGAATCCTCGATCGTCCGCACG	11	16	19	26	72	37.50	62.50
HAdV-D49	CTCGCGGTTGAGGGCGTACTCCTCGTCATCCTTCCAGTACTCCCGGAGCGGGAATCCTCGATCGTCCGCACG	10	16	20	26	72	36.11	63.89
HAdV-D51	CTCGCGGTTGAGGGCATACTCCTCGTCATCCTTCCAGTACTCCCGGAGCGGGAATCCTCGATCGTCCGCACG	11	16	19	26	72	37.50	62.50
HAdV-D53	CTCGCGGTTGAGAGCGTACTCCTCGTCATCCTTCCAGTACTCCCGGAGCGGGAATCCTCGATCGTCCGCACG	11	16	19	26	72	37.50	62.50
HAdV-D54	CTCGCGGTTAAGGGCGTACTCCTCGTCATCCTTCCAGTACTCCCGGAGCGGGAATCCTCGATCGTCCGCACG	11	16	19	26	72	37.50	62.50
HAdV-D56	CTCGCGGTTGAGGGCGTACTCCTCGTCATCCTTCCAGTACTCCCGGAGCGGGAATCCTCGATCGTCCGCACG	10	16	20	26	72	36.11	63.89
HAdV-D58	CTCGCGGTTGAGGGCGTACTCCTCGTCATCCTTCCAGTACTCCCGGAGCGGGAATCCTCGATCGTCCGCACG	10	16	20	26	72	36.11	63.89
HAdV-D59	CTCGCGGTTGAGGGCGTACTCCTCGTCATCCTTCCAGTACTCCCGGAGCGGGAATCCTCGATCGTCCGCACG	10	16	20	26	72	36.11	63.89
HAdV-D60	CTCGCGGTTGAGGGCGTACTCCTCGTCATCCTTCCAGTACTCCCGGAGCGGGAATCCTCGATCGTCCGCACG	10	16	20	26	72	36.11	63.89
HAdV-D62	CTCGCGGTTGAGGGCGTACTCCTCGTCATCCTTCCAGTACTCCCGGAGCGGGAATCCTCGATCGTCCGCACG	10	16	20	26	72	36.11	63.89
HAdV-D63	CTCGCGGTTGAGGGCGTACTCCTCGTCATCCTTCCAATACTCCCGGAGCGGGAATCCTCGATCGTCCGCACG	11	16	19	26	72	37.50	62.50
HAdV-D64	CGCGCGGTTGAGGGCGTACTCCTCGTCATCCTTCCAGTACTCCCGGAGCGGGAATCCTCGATCGTCCGCACG	10	15	21	26	72	34.72	65.28
HAdV-D65	CTCGCGGTTGAGGGCGTACTCCTCGTCATCCTTCCAGTACTCCCGGAGCGGGAATCCTCGATCGTCCGCACG	10	16	20	26	72	36.11	63.89

HAdV-D67	CTCGCGGTTGAGGGCGTACTCCTCGTCATCCTTCCAGTACTCCCGGAGCGGAAATCCTCGATCGTCCGCACG	11	16	19	26	72	37.50	62.50
HAdV-D69	CTCGCGGTTGAGGGCGTACTCCTCGTCATCCTTCCAGTACTCCCGGAGCGGGAATCCTCGATCGTCCGCACG	10	16	20	26	72	36.11	63.89
HAdV-D70	CTCGCGGTTGAGGGCGTACTCCTCGTCATCCTTCCAGTACTCCCGGAGCGGGAATCCTCGATCGTCCGCACG	10	16	20	26	72	36.11	63.89
HAdV-D71	CTCGCGGTTGAGGGCGTACTCCTCGTCATCCTTCCAGTACTCCCGGAGCGGGAATCCTCGATCGTCCGCACG	10	16	20	26	72	36.11	63.89
HAdV-D72	CTCGCGGTTGAGGGCATACTCCTCGTCATCCTTCCAGTACTCCCGGAGCGGGAATCCTCGATCGTCCGCACG	11	16	19	26	72	37.50	62.50
HAdV-E4	CTCGCGGTTGAGAAGGAACTCTTCGCGGTCCTTCCAGTACTCTTCAAGGGGGAACCCGTCCTGGTCGGCACG	12	16	22	22	72	38.89	61.11
HAdV-F40	TTCGCGGTTCAGGAGGTACTCCTCGCGATCCTTCCAGTAATCTTCGGCAGGAAAGCCACGCTCGTCTGCGCG	12	17	20	23	72	40.28	59.72
HAdV-F41	TTCGCGGTTCAGGAGGTACTCCTCGCGGTCCTTCCAGTAATCTTCGGCCGGAAAGCCACGTTCGTCTGCACG	11	18	20	23	72	40.28	59.72
HAdV-G52	TTCGCGATTGAGGAGGTATTCCTCGCGGTCTTTCCAGTACTCTTCGGCGGGAAATCCTTTTTCGTCCGCTCG	9	24	19	20	72	45.83	54.17

Nucleotide differences from the paradigm (first) type in each species shown in red.

Supplemental Table 5. Sequence and GC content for TPL3, by virus.

Type no.	TPL3	A	T	G	C	Sum	A/T	G/C
HAdV-A12	GAACTCCGCGCAAGGAGCTAAGCGTCTGCATATCCACCGGGTCGGAGAACCTTTCAAGAAAGGCATCTAGCCAGTCACAGTCACAAG	26	14	22	25	87	45.98	54.02
HAdV-A18	GAACTCCGCGCAAGGAGCTGAGTGTGTCCATATCCACCGGATCAGAAAATTTTTCAAGGAAGGCATCTAGCCAATCGCAGTCACAAG	27	17	21	22	87	50.57	49.43
HAdV-A31	AAACTCCGCGCCAGGACCTAAGCGACTGCATATCTACTGGGTCGGAAAACCTTTCAAGGAAGGCGTCAATCCAGTCACAGTCACAAG	27	15	20	25	87	48.28	51.72
HAdV-A61	GAACTCCGCGCCAGGACCTAAGCGACTGCATATCTACTGGGTCTGAAAACCTTTCAAGGAAGGCGTCAATCCAGTCACAGTCACAAG	26	16	20	25	87	48.28	51.72
HAdV-B3	GTACTCCGCGCAAGGACCTGATCGTCTCAAGATCCACGGGATCTGAAAACCTTTCGACGAAAGCGTCTAACCAGTCGCAATCGCAAG	25	16	20	26	87	47.13	52.87
HAdV-B7	GTACTCCGCGCAAGGACCTGATTGTCTCAAGATCCACGGGATCTGAAAACCTTTCGACGAAAGCGTCTAACCAGTCGCAATCGCAAG	25	17	20	25	87	48.28	51.72
HAdV-B11	GGACTGCACGCAGAGATCTGATCGTGTCAAGATCCACGGGATCTGAAAACCTTTCGACGAAAGCGTCTAACCAGTCACAGTCACAAG	27	16	21	23	87	49.43	50.57
HAdV-B21	GTACTCCGCGCAAGGACCTGATCGTCTCAAGATCCACGGGATCTGAAAACCTTTCGACGAAAGCGTCTAACCAGTCGCAATCGCAAG	25	16	20	26	87	47.13	52.87
HAdV-B14	GGACTGCGCGCAGAGATCTGATCGTGTCAAGATCCACGGGATCTGAAAACCTTTCAACGAAAGCGTCTAACCAGTCACAGTCACAAG	27	16	21	23	87	49.43	50.57
HAdV-B16	GTACTCCGCGCAAGGACCTGATCGTCTCAAGATCCACGGGATCTGAAAACCTTTCAACGAAAGCGTCTAACCAGTCGCAATCGCAAG	26	16	19	26	87	48.28	51.72
HAdV-B34	GGACTGCGCGCAGAGATCTGATCGTGTCAAGATCCACGGGATCTGAAAACCTTTCGACGAAAGCGTCTAACCAGTCACAGTCACAAG	26	16	22	23	87	48.28	51.72
HAdV-B35	GGACTGCGCGCAGAGATCTGATCGTGTCAAGATCCACGGGATCTGAAAACCTTTCGACGAAAGCGTCTAACCAGTCACAGTCACAAG	26	16	22	23	87	48.28	51.72
HAdV-B50	GTACTCCGCGCAAGGACCTGATCGTCTCAAGATCCACGGGATCTGAAAACCTTTCGACGAAAGCGTCTAACCAGTCGCAATCGCAAG	25	16	20	26	87	47.13	52.87
HAdV-B55	GGACTGCGCGCAGAGATCTGATCGTGTCAAGATCCACGGGATCTGAAAACCTTTCAACGAAAGCGTCTAACCAGTCACAGTCACAAG	27	16	21	23	87	49.43	50.57
HAdV-B66	GTACTCCGCGCAAGGACCTGATCGTTTCAAGATCCACGGGATCTGAAAACCTTTCGACGAAAGCGTCTAACCAGTCGCAATCGCAAG	25	17	20	25	87	48.28	51.72
HAdV-B68	GTACTCCGCGCAAGGACCTGATCGTCTCAAGATCCACGGGATCTGAAAACCTTTCGACGAAAGCGTCTAACCAGTCGCAATCGCAAG	25	16	20	26	87	47.13	52.87
HAdV-C1	GTACTCCGCCACCGAGGGACCTGAGCGAGTCCGCATCGACCGGATCGGAAAACCTCTCGAGAAAGGCGTCTAACCAGTCACAGTCGCAAG	24	12	25	29	90	40.00	60.00
HAdV-C2	GTACTCCGCCACCGAGGGACCTGAGCGAGTCCGCATCGACCGGATCGGAAAACCTCTCGAGAAAGGCGTCTAACCAGTCACAGTCGCAAG	24	12	25	29	90	40.00	60.00
HAdV-C5	GTACTCCGCCGCCGAGGGACCTGAGCGAGTCCGCATCGACCGGATCGGAAAACCTCTCGAGAAAGGCGTCTAACCAGTCACAGTCGCAAG	23	12	26	29	90	38.89	61.11
HAdV-C6	GTACTCCGCCGCCGAGGGACCTGAGCGAGTCCGCATCGACCGGATCGGAAAACCTCTCGAGAAAGGCGTCTAACCAGTCACAGTCGCAAG	23	12	26	29	90	38.89	61.11
HAdV-C57	GTACTCCGCCACCGAGGGACCTGAGCGAGTCCGCATCGACCGGATCGGAAAACCTCTCGAGAAAGGCGTCTAACCAGTCACAGTCGCAAG	24	12	25	29	90	40.00	60.00
HAdV-D8	GGGACGTAAGCACGTCAAGATCGACCGGATCGGAAAATTTTTGAAGGAAAGCGTCTAGCCAATCGCAGTCACAAG	25	13	21	16	75	50.67	49.33
HAdV-D9	GGGACGTGAGCGCGTCGAGATCGACCGGATCGGAGAATCTTTCGAGGAAAGCGTCTAGCCAATCGCAGTCGCAAG	19	12	26	18	75	41.33	58.67
HAdV-D10	GGGACGTGAGCGCGTCGAGATCGACCGGATCGGAGAATCTTTCGAGGAAAGCGTCTAGCCAATCGCAGTCGCAAG	19	12	26	18	75	41.33	58.67
HAdV-D13	GGGACGTGAGCGCGTCGAGATCGACCGGATCGGAGAATCTTTCGAGGAAAGCGTCTAGCCAATCGCAGTCGCAAG	19	12	26	18	75	41.33	58.67
HAdV-D15	GGGACGTGAGAGCGTCGAGATCGACCGGATCGGAGAATCTTTCGAGGAAAGCGTCTAGCCAATCGCAGTCGCAAG	20	12	26	17	75	42.67	57.33
HAdV-D17	GGGACGTGAGCGCGTCGAGATCGACCGGATCGGAGAATCTTTCGAGGAAAGCGTCTAGCCAATCGCAGTCGCAAG	19	12	26	18	75	41.33	58.67
HAdV-D19	GGGACGTGAGCGCGTCGAGATCGACCGGATCGGAGAATCTTTCGAGGAAAGCGTCTAGCCAATCGCAGTCGCAAG	19	12	27	17	75	41.33	58.67
HAdV-D20	GGGACGTGAGCGCGTCGAGATCGACCGGATCGGAGAATCTTTCGAGGAAAGCGTCTAGCCAATCGCAGTCGCAAG	19	12	26	18	75	41.33	58.67

HAdV-D22	GGGACGTGAGCGCGTCGAGATCGACCGGATCGGAGAATCTTTCGAGGAAAGCGTCTAGCCAATCGCAGTCGCAAG	19	12	26	18	75	41.33	58.67
HAdV-D23	GGGACGTGAGCGCGTCGAGATCGACCGGATCGGAGAATCTTTCGAGGAAAGCGTCTAGCCAATCGCAGTCGCAAG	19	12	26	18	75	41.33	58.67
HAdV-D24	GGGACGTGAGCGCGTCGAGATCGACCGGATCGGAGAATCTTTCGAGGAAAGCGTCTAGCCAATCGCAGTCGCAAG	19	12	26	18	75	41.33	58.67
HAdV-D25	GGGACGTGAGCGCGTCGAGATCGACCGGATCGGAGAATCTTTCGAGGAAAGCGTCTAGCCAATCGCAGTCGCAAG	19	12	26	18	75	41.33	58.67
HAdV-D26	GGGACGTGAGCGCGTCGAGATCGACCGGATCGGAGAATCTTTCGAGGAAAGCGTCTAGCCAATCGCAGTCGCAAG	19	12	26	18	75	41.33	58.67
HAdV-D27	GGGACGTGAGCGCGTCGAGATCGACCGGATCGGAGAATCTTTCGAGGAAAGCGTCTAGCCAATCGCAGTCGCAAG	19	12	26	18	75	41.33	58.67
HAdV-D28	GGGACGTGAGCGCGTCGAGATCGACCGGATCGGAGAATCTTTCGAGGAAAGCGTCTAGCCAATCGCAGTCGCAAG	19	12	26	18	75	41.33	58.67
HAdV-D29	GGGACGTGAGCGCGTCGAGATCGACCGGATCGGAGAATCTTTCGAGGAAAGCGTCTAGCCAATCGCAGTCGCAAG	19	12	26	18	75	41.33	58.67
HAdV-D30	GGGACGTGAGCGCGTCGAGATCGACCGGATCGGAGAATCTTTCGAGGAAAGCGTCTAGCCAATCGCAGTCGCAAG	19	12	26	18	75	41.33	58.67
HAdV-D32	GGGACGTGAGCGCGTCGAGATCGACCGGATCGGAGAATCTTTCGAGGAAAGCGTCTAGCCAATCGCAGTCGCAAG	19	12	26	18	75	41.33	58.67
HAdV-D33	GGGACGTGAGCGCGTCGAGATCGACCGGATCGGAGAATCTTTCGAGGAAAGCGTCTAGCCAATCGCAGTCGCAAG	19	12	26	18	75	41.33	58.67
HAdV-D36	GGGACGTGAGCGCGTCGAGATCGACCGGATCGGAGAATCTTTCGAGGAAAGCGTCTAGCCAATCGCAGTCGCAAG	19	12	26	18	75	41.33	58.67
HAdV-D37	GGCACGTGAGCGCGTCGAGATCGACCGGATCGGAGAATCTTTCGAGGAAAGCGTCTAGCCAATCGCAGTCGCAAG	19	12	25	19	75	41.33	58.67
HAdV-D38	GGGACGTGAGCGCGTCGAGATCGACCGGATCGGAGAATCTTTCGAGGAAAGCGTCTAGCCAATCGCAGTCGCAAG	19	12	26	18	75	41.33	58.67
HAdV-D39	GGGACGTGAGCGCGTCGAGATCGACCGGATCGGAGAATCTTTCGAGGAAAGCGTCTAGCCAATCGCAGTCGCAAG	19	12	26	18	75	41.33	58.67
HAdV-D42	GGGACGTGAGCGCGTCGAGATCGACCGGATCGGAGAATCTTTCGAGGAAAGCGTCTAGCCAATCGCAGTCGCAAG	19	12	26	18	75	41.33	58.67
HAdV-D43	GGGACGTGAGCGCGTCGAGATCGACCGGATCGGAGAATCTTTCGAGGAAAGCGTCTAGCCAATCGCAGTCGCAAG	19	12	26	18	75	41.33	58.67
HAdV-D44	GGGACGTGAGCGCGTCGAGATCGACCGGATCGGAGAATCTTTCGAGGAAAGCGTCTAGCCAATCGCAGTCGCAAG	19	12	26	18	75	41.33	58.67
HAdV-D45	GGGACGTGAGCGCGTCGAGATCTACCGGATCGGAGAATCTTTCGAGGAAAGCGTCTAGCCAATCGCAGTCGCAAG	19	13	25	18	75	42.67	57.33
HAdV-D46	GGGACGTGAGCGCGTCGAGATCGACCGGATCGGAAAATCTTTCGAGGAAAGCGTCTAGCCAATCGCAGTCGCAAG	20	12	25	18	75	42.67	57.33
HAdV-D47	GGGACGTGAGCGCGTCGAGATCGACCGGATCGGAGAATCTTTCGAGGAAAGCGTCTAGCCAATCGCAGTCGCAAG	19	12	26	18	75	41.33	58.67
HAdV-D48	GGGACGTGAGCGCGTCGAGATCGACCGGATCGGAGAATCTTTCGAGGAAAGCGTCTAGCCAATCGCAGTCGCAAG	19	12	26	18	75	41.33	58.67
HAdV-D49	GGGACGTGAGCGCGTCGAGATCGACCGGATCGGAGAATCTTTCGAGGAAAGCGTCTAGCCAATCGCAGTCGCAAG	19	12	26	18	75	41.33	58.67
HAdV-D51	GGGACGTGAGCGCGTCGAGATCGACCGGATCGGAGAATCTTTCGAGGAAAGCGTCTAGCCAATCGCAGTCGCAAG	19	12	26	18	75	41.33	58.67
HAdV-D53	GGCACGTGAGCGCGTCGAGATCGACCGGATCGGAGAATCTTTCGAGGAAAGCGTCTAGCCAATCGCAGTCGCAAG	19	12	25	19	75	41.33	58.67
HAdV-D54	GGGACGTAAGCGCGTCAAGATCGACCGGATCGGAGAATCTTTGGAGGAAAGCGTCTAGCCAATCGCAGTCGCAAG	21	12	25	17	75	44.00	56.00
HAdV-D56	GGGACGTGAGCGCGTCGAGATCGACCGGATCGGAGAATCTTTCGAGGAAAGCGTCTAGCCAATCGCAGTCGCAAG	19	12	26	18	75	41.33	58.67
HAdV-D58	GGGACGTGAGCGCGTCGAGATCGACCGGATCGGAGAATCTTTCGAGGAAAGCGTCTAGCCAATCGCAGTCGCAAG	19	12	26	18	75	41.33	58.67
HAdV-D59	GGGACGTGAGCGCGTCGAGATCGACCGGATCGGAGAATCTTTCGAGGAAAGCGTCTAGCCAATCGCAGTCGCAAG	19	12	26	18	75	41.33	58.67
HAdV-D60	GGGACGTGAGTGCGTCGAGATCGACCGGATCGGAGAATCTTTCGAGGAAAGCGTCTAGCCAATCGCAGTCGCAAG	19	13	26	17	75	42.67	57.33
HAdV-D62	GGGACGTGAGCGCGTCGAGATCGACCGGATCGGAGAATCTTTCGAGGAAAGCGTCTAGCCAATCGCAGTCGCAAG	19	12	26	18	75	41.33	58.67
HAdV-D63	GGGACGTGAGCGCGTCGAGATCGACCGGATCGGAGAATCTTTCGAGGAAAGCGTCTAGCCAATCGCAGTCGCAAG	19	12	26	18	75	41.33	58.67
HAdV-D64	GGCACGTGAGCGCGTCGAGATCGACCGGATCGGAGAATCTTTCGAGGAAAGCGTCTAGCCAATCGCAGTCGCAAG	19	12	25	19	75	41.33	58.67
HAdV-D65	GGGACGTGAGCGCGTCGAGATCGACCGGATCGGAGAATCTTTCGAGGAAAGCGTCTAGCCAATCGCAGTCGCAAG	19	12	26	18	75	41.33	58.67

HAdV-D67	GGGACGTGAGCGCGTCGAGATCGACCGGATCGGAGAATCTTTCGAGGAAAGCGTCTAGCCAATCGCAGTCGCAAG	19	12	26	18	75	41.33	58.67
HAdV-D69	GGGACGTGAGCGCGTCGAGATCGACCGGATCGGAGAATCTTTCGAGGAAAGCGTCTAGCCAATCGCAGTCGCAAG	19	12	26	18	75	41.33	58.67
HAdV-D70	GGGACGTGAGCGCGTCGAGATCGACCGGATCGGAGAATCTTTCAAGGAAAGCGTCTAGCCAATCGCAGTCGCAAG	20	12	25	18	75	42.67	57.33
HAdV-D71	GGGACGTGAGCGCGTCGAGATCGACCGGATCGGAGAATCTTTCGAGGAAAGCGTCTAGCCAATCGCAGTCGCAAG	19	12	26	18	75	41.33	58.67
HAdV-D72	GGGACGTGAGCGCGTCGAGATCGACCGGATCGGAGAATCTTTCGAGGAAAGCGTCTAGCCAATCGCAGTCGCAAG	19	12	26	18	75	41.33	58.67
HAdV-E4	GGACTCCGCGCAAGGACCTAAGCGTCTCTAGATCCACGGGATCTGAAAACCGTTGAACGAAGGCTTCGAGCCAGTCGCAGTCGCAAG	23	14	25	25	87	42.53	57.47
HAdV-F40	GTAGAGACTGCCAAGCCTCATTGAGCGAGTCCAGTTGGACGGGATCAGAGAATTTTTCGAGGAAAGCTTCCAGCCAATCGCAGTCGCAAG	25	18	26	21	90	47.78	52.22
HAdV-F41	GCAGAGATTGCCAAGCGTTGTTGAGCAAGTCCAATTGGACGGGATCAGAAAACTTTTCAAGGAAAGCATCGAGCCAATCACAGTCGCAAG	30	17	24	19	90	52.22	47.78
HAdV-G52	GCACTCCGTGCAGGGATCTGAGAACGTCGAGGTCGACGGGATCCGAGAACTTCTCTAGGAAAGCGTCTATCCAATCGCAATCGCAAG	23	16	25	23	87	44.83	55.17

Nucleotide differences from the paradigm (first) type in each species shown in red.

Supplemental Table 6. i-leader start, end, and splice sites for representative viruses of each HAdV species.

Species and type of virus	i-leader			i-leader to TPL3 start (ATG)/ splice (end)					i-protein mRNA (unspliced)		
	**Start**	**End**	**Length**	**Start**	**End**	**Length**	**TPL3**	**Sum**	**Start**	**End**	**Length**
HAdV-A12	7705	7730	26	7731	8138	408	21	429	7731	8219	489
HAdV-B3	7803	7828	26	7829	8227	399	21	420	7829	8425	597
HAdV-B11	7819	7844	26	7845	8243	399	21	420	7845	8255	411
HAdV-C2	7952	7977	26	7978	8391	414	24	438	7978	8427	450
HAdV-D37	7760	7785	26	7786	8166	381	9	390	7786	8253	468
HAdV-E4	7788	7813	26	7814	8212	399	21	420	7814	8407	594
HAdV-F40	7510	7535	26	7536	8088	553	24	577	7536	8226	691
HAdV-G52	7436	7450	15	7478	7686	209	21	230	7478	7636	159

Supplemental Table 7. Putative j-leader start and end sites, identity comparisons to HAdV-D37, and genomic sequences in HAdV-D.

Name	j-leader Start	j-leader End	Portions	Identities (%) (vs HAdV-D37)	Sequence
HAdV-D37	26764	26889	126/126	100.0	AGGGAACCTACCAGTGCCAGAGCGGACCTTGCTTCCACAGTTTTACTTTGGTGAACGTTACCGGCAGCAGCACAGCCGCTCCAGAAACTAACCTTCCTTCTGATACTATCAAACCTTGTTTCGGAG
HAdV-D64	26781	26906	126/126	100.0	AGGGAACCTACCAGTGCCAGAGCGGACCTTGCTTCCACAGTTTTACTTTGGTGAACGTTACCGGCAGCAGCACAGCCGCTCCAGAAACTAACCTTCCTTCTGATACTATCAAACCTTGTTTCGGAG
HAdV-D9	26750	26875	123/126	97.6	AGGGAACCTACCACTGCCAGAGCGGACCTTGCTTCCACAGTTTTACTTTGGTGAACGTTACCGGCAGCAGCACAGCCGCTCCAGAACCTAACCTTCCTTCTGATACTATCAAACCTCGTTTCGGAG
HAdV-D10	26735	26860	123/126	97.6	AGGGAACCTACCACTGCCAGAGCGGACCTTGCTTCCACAGTTTTACTTTGGTGAACGTTACCGGCAGCAGCACAGCCGCTCCAGAACCTAACCTTCCTTCTGATACTATCAAACCTCGTTTCGGAG
HAdV-D15	26772	26897	121/126	96.0	AGGGAACCTACCAGTGCCTGAGTGGACCTTGCTTCCACAGTTTTACTTTGGTGAACGTTACCGACAGCAGCACAGCCGCTCCAGAAACTAACCTTCCTTCTGATACTAACAAACCTCGTTTCGGAG
HAdV-D60	26679	26804	121/126	96.0	AGGGAACCTACCAGTGCCTGAGTGGACCTTGCTTCCACAGTTTTACTTTGGTGAACGTTACCGACAGCAGCACAGCCGCTCCAGAAACTAACCTTCCTTCTGATACTAACAAACCTCGTTTCGGAG
HAdV-D48	26750	26875	119/126	94.4	AAGGAACTTACCAGTGTCAGAGCGGACCTTGCTTCCACACTTTCACTTTGGTGAACGTTACCGGCAGCAGCACAGCCGCTCCAGAAACTAACCTTCCTTCTGATACTAACAAACCTCGTTTCGGAG
HAdV-D24	26720	26848	118/129	91.5	AAGGAACTTACCAGTGTCAGAGCGGACCTTGCTTCCACAGTTTCACTTTGGTGAACGTTACCGGCAGCAGCACAGCCGCTCCAGAAACATCTAACCTTCTTTCTGATACTAACAAACCTCGTGTCGGAG
HAdV-D46	26732	26860	118/129	91.5	AAGGAACTTACCAGTGTCAGAGCGGACCTTGCTTCCACAGTTTCACTTTGGTGAACGTTACCGGCAGCAGCACAGCCGCTCCAGAAACATCTAACCTTCTTTCTGATACTAACAAACCTCGTGTCGGAG
HAdV-D13	26768	26896	115/129	89.1	AGGGAACCTACCACTGCAAGAGCGGATCTTGCTACCACACTTTCACTTTGGTGAACGTTACCGGCAGCAGCACAGCCGCTCCTGAAACAACTAACCTTTCTTCTGATACTAACAAACCTCGTGTCGGAG
HAdV-D47	26779	26907	111/129	86.0	AAGGAACTTATCACTGCCAGAGCGGAGGTTGCTACCACAGTTTCACTTTGGTGAACGTTACCAGCAGCAGTACAACCGCTCCAGAAACAACTAACCTTTCTTCTGATACTAACAAACCTCATTTCGGAG
HAdV-D56	26716	26841	116/135	85.9	AGGGAACCTACCAGTGCCAGAGCGGACCTTGTACTCACACCTTCCA---------TTTGGTGAACGTTACCGGCAGCAGCACAGCCGCTCCAGAAACTAACCTTCCTTCTGATACTATCAAACCTCGTTTCGGAG
HAdV-D33	26743	26868	107/127	84.3	AGGGAACCTACCAGTGCC-GACACGGACCTTGTGTTCACAACTTCCATTTGGTGAACCTTACCAGCAGCAGCACAGTTGCTCCTGAAACTAACCTTTCTTCTGATACTAACAAACCTCGTGTCGGAG
HAdV-D43	26688	26813	107/127	84.3	AGGGAACCTACCAGTGCC-GACACGGACCTTGTGTTCACAACTTCCATTTGGTGAACCTTACCAGCAGCAGCACAGTTGCTCCTGAAACTAACCTTTCTTCTGATACTAACAAACCTCGTGTCGGAG
HAdV-D36	26739	26864	112/133	84.2	AAGGAACTTACCAGTGTCAGAGCGGACCTTGCTTCCACAGTTTCACTTTGGTGAACGTTACCGGCAGCAGCACAGTCGCTCCAGAAACTAACCTTCTTTCTGATACTAACACTCCTAAAAC-------CGGAG
HAdV-D29	26754	26882	114/136	83.8	AGGGAACCTACCACTGCCAGAGCGGACCTTGCTTCCACACTTTCACTTTGGTGAACGTTACCGGCAGCAGCACAGCCGCTCCAGAAACATCTAACCTTCTTTCTGATACTAACACTCCTAAAAC-------CGGAG
HAdV-D63	26718	26846	114/136	83.8	AGGGAACCTACCACTGCCAGAGCGGACCTTGCTTCCACACTTTCACTTTGGTGAACGTTACCGGCAGCAGCACAGCCGCTCCAGAAACATCTAACCTTCTTTCTGATACTAACACTCCTAAAAC-------CGGAG
HAdV-D27	26714	26839	106/127	83.5	AGGGAACCTACCAGTGCC-GACACGGACCTTGTGTTCACAACTTCCATTTGGTGAACCTTACCAGCAGCAGCACAGTTGCTCCTGAAAGTAACCTTTCTTCTGATACTAACAAACCTCGTGTCGGAG
HAdV-D32	26767	26892	106/127	83.5	AGGGAACCTACCAGTGCC-GACACGGACCTTGTGTTCACAACTTCCATTTGGTGAACCTTACCAGCAGCAGCACAGTTGCTCCTGAAAGTAACCTTTCTTCTGATACTAACAAACCTCGTGTCGGAG
HAdV-D38	26756	26884	110/132	83.3	AGGGAACCTACCAGTGCC-GACACGGACCTTGTGTTCACACA-TTCCAC-TTGGTGAACCTTACCAGCAGCAGCACAGTTGCTCCTGAAACAACTAACCTTTCTTCTGATACTAACAAACCTCGTGTCGGAG
HAdV-D49	26760	26888	110/132	83.3	AGGGAACCTACCAGTGCC-GACACGGACCTTGTGTTCACACA-TTCCAC-TTGGTGAACCTTACCAGCAGCAGCACAGTTGCTCCTGAAACAACTAACCTTTCTTCTGATACTAACAAACCTCGTGTCGGAG
HAdV-D71	26778	26906	110/132	83.3	AGGGAACCTACCAGTGCC-GACACGGACCTTGTGTTCACACA-TTCCAC-TTGGTGAACCTTACCAGCAGCAGCACAGTTGCTCCTGAAACAACTAACCTTTCTTCTGATACTAACAAACCTCGTGTCGGAG
HAdV-D72	26735	26863	110/132	83.3	AGGGAACCTACCAGTGCC-GACACGGACCTTGTGTTCACACA-TTCCAC-TTGGTGAACCTTACCAGCAGCAGCACAGTTGCTCCTGAAACAACTAACCTTTCTTCTGATACTAACAAACCTCGTGTCGGAG
HAdV-D20	26758	26886	113/136	83.1	AGGGAACCTACCACTGCCAGAGCGGACCTTGCTTCCACACTTTCACTTTGGTGAACGTTACCGACAGCAGCACAGCCGCTCCAGAAACATCTAACCTTCTTTCTGATACTAACACTCCTAAAAC-------CGGAG
HAdV-D22	26731	26859	113/136	83.1	AGGGAACCTACCACTGCCAGAGCGGACCTTGCTTCCACACTTTCACTTTGGTGAACGTTACCGACAGCAGCACAGCCGCTCCAGAAACATCTAACCTTCTTTCTGATACTTTCACTCCTAAAAC-------CGGAG
HAdV-D44	26742	26870	113/136	83.1	AGGGAACCTACCACTGCCAGAGCGGACCTTGCTACCACACTTTCACTTTGGTGAACGTTACCGGCAGCAGCACAGCCGCTCCAGAAACATCTAACCTTCTTTCTGATACTAACACTCCTAAAAC-------CGGAG
HAdV-D45	26763	26891	113/136	83.1	AGGGAACCTACCACTGCCAGAGCGGACCTTGCTACCACACTTTCACTTTGGTGAACGTTACCGGCAGCAGCACAGCCGCTCCAGAAACATCTAACCTTCTTTCTGATACTAACACTCCTAAAAC-------CGGAG
HAdV-D23	26703	26831	112/136	82.4	AGGGAACCTACCACTGCCAGAGCGGACCTTGCTTCCACACTTTCACTTTGGTGAACGTTACCGACAGCAGCACAGCCGCTCCAGAAACATCTAACCTTCTTTCTGAAACTAACACTCCTAAAAC-------CGGAG
HAdV-D25	26746	26874	112/136	82.4	AGGGAACCTACCACTGCCAGAGCGGACCTTGCTACCACACTTTCACTTTGGTGAACGTTACCGGCAGCAGCACAGCCGCTCCAGAAACATCTAACCTTCTTTTTGATACTAACACTCCTAAAAC-------CGGAG

HAdV-D42	26781	26909	112/136	82.4	AGGGAACCTACCACTGCCAGAGCGGACCTTGCTTCCACACTTTCACTTTGGTGAACGTTAACGACAGCAGCACAGCCGCTCCAGAAACATCTAACCTTCTTTCTGATACTAACACTCCTAAAAC-------CGGAG
HAdV-D17	26754	26882	111/136	81.6	AGGGAACCTACCACTGCCAGAGCGGACCTTGCTTCCACACTTTCACTTTGGTGAACGTTACCGACAGCAGCACAGCCGCTACAGAAACATCTAACCTTCTTTTTGATACTAACACTCCTAAAACCGGAG
HAdV-D28	26743	26871	106/130	81.5	AGGGAACCTACCAGTGCC-GACACGGACCTTGTGTTCACACATTCCAATTGGTGAACCTTACCAGCAGCAGCACAGTTGCTCCTGAAACAACTAACCTTTCTTCTGATACTAACAAACCTCGTGTCGGAG
HAdV-D30	26711	26839	106/130	81.5	AGGGAACCTACCAGTGCC-GACACGGACCTTGTGTTCACAACTTCCATTTGGTGAACCTTACCAGCAGCAGTACAGTTGCTCCTGAAACAACTAACCTTTCTTCTGATACTAACAAACCTCGTGTCGGAG
HAdV-D39	26763	26891	106/130	81.5	AGGGAACCTACCAGTGCC-GACACGGACCTTGTGTTCACAACTTCCATTTGGTGAACCTTACCAGCAGCAGTACAGTTGCTCCTGAAACAACTAACCTTTCTTCTGATACTAACAAACCTCGTGTCGGAG
HAdV-D62	26750	26878	106/130	81.5	AGGGAACCTACCAGTGCC-GACACGGACCTTGTGTTCACAACTTCCATTTGGTGAACCTTACCAGCAGCAGTACAGTTGCTCCTGAAACAACTAACCTTTCTTCTGATACTAACAAACCTCGTGTCGGAG
HAdV-D53	26696	26821	110/136	80.9	AGGGAACCTACCACTGCCAGAGCGGACCTTGCTTCCACACTTTCACTTTGGTGAACGTTACCGACAGCAGCACAGCCGCTCCAGAAACATCTAACCT---TTTTGATACTAACACTCCTAAAAC-------CGGAG
HAdV-D58	26776	26904	105/130	80.8	AGGGAACCTACCAGTGCC-GACACGGACCCTGTGTTCACAACTTCCATTTGGTGAACCTTACCAGCAGCAGTACAGTTGCTCCTGAAACAACTAACCTTTCTTCTGATACTAACAAACCTCGTGTCGGAG
HAdV-D8	26697	26818	103/128	80.5	AAGGAACTTACCATTGTCAGAGCGGACCTTGTCACCACA-TTTTCCATTTGGTGAACGTCACCAGCAGCAGCAACA---GCTCC-GAAACTAACCTTTCTTCTCGTACTAACAGACCTAATTT-GGAG
HAdV-D59	26763	26888	109/136	80.1	AGGGAACCTACCACTGCCAGAGCGGACCTTGCTTCCACACTTTCACTTTGGTGAACGTTACCGACAGCAGCGCAGCCGCTCCAGAAACATCTAACCT---TTTTGATACTAACACTCCTAAAAC-------CGGAG
HAdV-D69	26730	26855	109/136	80.1	AGGGAACCTACCACTGCCAGAGCGGACCTTGCTTCCACACTTTCACTTTGGTGAACGTTACCGACAGCAGCACAGTCGCTCCAGAAACATCTAACCT---TTTTGATACTAACACTCCTAAAAC-------CGGAG
HAdV-D26	26778	26900	108/136	79.4	AGGGAACCTACCAGTGCCAGAGCGGACCTTGTACTCACACCTTCCA---------TTTGGTGAACGTCACCAGCAGCAGCAACAGC----TCAGAAACTAACCTTCCTTCTGATACTAACAAACCTCGTTTCGGAG
HAdV-D19	26767	26889	101/128	78.9	AAGGAACTTACCATTGTCAGAGCGGACCTTGTCACCACA-TTTTCCATTTGGTGAACGTCACCAGCAGCAGCAACAGC----TCAGAAACTAACCTCTCTTCTCGTACTAACAGACCTCAATTCGGAG
HAdV-D51	26770	26892	101/128	78.9	AAGGAACTTACCATTGTCAGAGCGGACCTTGTCACCACA-TTTTCCATTTGGTGAACGTCACCAGCAGCAGCAACAGC----TCAGAAACTAACCTCTCTTCTCGTACTAACAGACCTCAATTCGGAG
HAdV-D54	26623	26745	102/130	78.5	AAGGAACTTACCATTGTCAGAGCGGACCTTG--TCATCACA-TTTTCCATTTGGTGAACGTCACCAGCAGCAGCAACAGC----TCAGAAACTAACCTCTCTTCTCGTACTAACAGACCTCAATTCGGAG
HAdV-D65	26770	26889	98/128	76.6	AAGGAACTTACCATTGTCACAGCGGACCTTGTCACCACA-TTTTCCATTTGGTGAACGTCACCAGCAGCAGCAACAGC----TCAGAAACTAACCT---CTCTCGTACTAACAGACCTCAATTCGGAG
HAdV-D70	26736	26864	105/139	75.5	AGGGAACCTACCAGTGCC-GACACGGACCTTGTGTTCTCAACTTCCA---------TTTGGTGAACATTACCAGCAGCAGTACAGTTGCTCCTGAAACAACTAACCTTTCTTCTGATACTAACAAACCTCGTGTCGGAG
HAdV-D67	26738	26860	99/136	72.8	AGGGAACCTATCATTGCCAGAGCGGACCTTGTACTCACACCTTCCA---------TTTGGTGAACGTCACCAGCAGCAGCAACAGC----TCAGAAACTAACCTCTCTTCTCGTACTAACAGACCTCATTTTGGAG

Supplemental Table 8. Comparison of individual TPLs with BPLs.

TPL1 vs. BPL1 vs. BPL2

Virus species and type	HAdV-A12	HAdV-B3	HAdV-B11	HAdV-C2	HAdV-D37	HAdV-E4	HAdV-F40	HAdV-G52	MAV2-BPL1	MAV2-BPL2
HAdV-A12	100.00									
HAdV-B3	73.17	100.00								
HAdV-B11	68.29	95.12	100.00							
HAdV-C2	75.61	85.37	80.49	100.00						
HAdV-D37	78.05	90.24	85.37	90.24	100.00					
HAdV-E4	73.17	100.00	95.12	85.37	90.24	100.00				
HAdV-F40	75.61	80.49	78.05	82.93	82.93	80.49	100.00			
HAdV-G52	75.61	80.49	75.61	82.93	82.93	80.49	95.12	100.00		
MAV2-BPL1	51.22	48.78	46.34	48.78	48.78	48.78	56.10	56.10	100.00	
MAV2-BPL2	53.66	53.66	53.66	53.66	53.66	53.66	56.10	51.22	50.00	100.00

TPL2 vs. BPL1 vs. BPL2

Virus species and type	HAdV-A12	HAdV-B3	HAdV-B11	HAdV-C2	HAdV-D37	HAdV-E4	HAdV-F40	HAdV-G52	MAV2-BPL1	MAV2-BPL2
HAdV-A12	100.00									
HAdV-B3	77.78	100.00								
HAdV-B11	76.39	93.06	100.00							
HAdV-C2	63.89	76.39	76.39	100.00						
HAdV-D37	66.67	72.22	70.83	70.83	100.00					
HAdV-E4	68.06	86.11	86.11	79.17	70.83	100.00				
HAdV-F40	70.83	75.00	77.78	68.06	72.22	73.61	100.00			
HAdV-G52	75.00	77.78	79.17	73.61	70.83	75.00	80.56	100.00		
MAV2-BPL1	32.79	37.70	34.43	36.07	40.98	36.07	34.43	37.70	100.00	
MAV2-BPL2	37.50	43.06	43.06	41.67	50.00	44.44	38.89	33.33	43.21	100.00

TPL3 vs. BPL1 vs. BPL2

**Virus species and type**	**HAdV-A12**	**HAdV-B3**	**HAdV-B11**	**HAdV-C2**	**HAdV-D37**	**HAdV-E4**	**HAdV-F40**	**HAdV-G52**	**MAV2-BPL1**	**MAV2-BPL2**
HAdV-A12	100.00									
HAdV-B3	77.01	100.00								
HAdV-B11	74.71	88.51	100.00							
HAdV-C2	73.56	74.71	74.71	100.00						
HAdV-D37	73.33	78.67	77.33	81.33	100.00					
HAdV-E4	80.46	86.21	79.31	68.97	73.33	100.00				
HAdV-F40	58.62	62.07	62.07	62.22	80.00	60.92	100.00			
HAdV-G52	67.82	77.01	73.56	68.97	81.33	70.11	64.37	100.00		
MAV2-BPL1	29.63	33.33	37.04	33.33	33.33	35.19	37.04	38.89	100.00	
MAV2-BPL2	39.08	45.98	43.68	40.00	36.00	43.68	36.67	39.08	53.66	100.00

Supplemental Table 9. Similarity of entire TPL without and with i-leader included, with entire BPL.

TPL1-3 vs BPL1-2

**Virus species and type**	**HAdV-A12**	**HAdV-B3**	**HAdV-B11**	**HAdV-C2**	**HAdV-D37**	**HAdV-E4**	**HAdV-F40**	**HAdV-G52**	**MAV-2**
HAdV-A12	100.00								
HAdV-B3	76.50	100.00							
HAdV-B11	74.00	91.50	100.00						
HAdV-C2	72.50	79.50	77.50	100.00					
HAdV-D37	70.81	77.84	75.68	78.19	100.00				
HAdV-E4	74.50	89.00	85.00	78.00	75.14	100.00			
HAdV-F40	67.00	72.00	72.50	68.47	77.66	70.50	100.00		
HAdV-G52	72.00	78.00	76.00	75.50	77.30	74.00	78.50	100.00	
MAV-2	41.24	45.88	45.36	42.86	41.99	45.88	41.84	41.75	100.00

TPL1-3 with i-leader(26nt) vs BPL1-2

**Virus species and type**	**HAdV-A12**	**HAdV-B3**	**HAdV-B11**	**HAdV-C2**	**HAdV-D37**	**HAdV-E4**	**HAdV-F40**	**HAdV-G52**	**MAV-2**
HAdV-A12	100.00								
HAdV-B3	76.11	100.00							
HAdV-B11	73.45	91.15	100.00						
HAdV-C2	66.81	74.34	72.57	100.00					
HAdV-D37	66.35	72.99	71.09	75.70	100.00				
HAdV-E4	73.89	89.38	85.40	72.57	70.62	100.00			
HAdV-F40	61.50	66.81	66.81	65.07	71.50	66.37	100.00		
HAdV-G52	69.30	75.35	73.49	73.49	72.46	71.63	73.02	100.00	
MAV-2	40.98	41.95	40.49	42.31	42.56	40.98	39.42	44.95	100.00

Supplemental Table 10. PCR primers applied in the identification of 5'UTR leader sequences for HAdV-D37.

Sequence Name	Primer sequence	Start	End
HAdV-D37_52/55K_R	GCCCCCAGTCTCGCCA	10780	10765
HAdV-D37_pIIIa_R	CGCCGCCCTTATGGCGGG	11820	11803
HAdV-D37_Penton_R	AGCCATATACCGCGGAGGCACAAA	13631	13608
HAdV-D37_pVII_R	GGTGCTGCTGGGAGCGTT	15189	15172
HAdV-D37_pV_R	GCACAAACTCTACTGCCCCCTC	15860	15839
HAdV-D37_pX_R	GGATGAGCGCGGGCAGAAAG	16900	16881
HAdV-D37_pVI_R	TTAAGCCCGCTCCAGACACTGC	17163	17142
HAdV-D37_Hexon_R	GTCTAGCACCCGGTTGTCGC	18089	18070
HAdV-D37_Protease_R	GCCGGCCGTGTTGACGAT	20770	20753
HAdV-D37_100K_R	CCTCCTGGCCTGCGTCT	22944	22928
HAdV-D37_22K_R	TGGTGCGGCGACGGGCTG	24927	24910
HAdV-D37_pVIII_R	CGGACCCCGTTGACCCG	25656	25640
HAdV-D37_12.0K_R	AACCGAGCGAGCTCAGCCGAGT	26232	26211
HAdV-D37_21.8K_R	AGTCTGGTTGGTGAGGTAGAC	26585	26565
HAdV-D37_18.6K_R	ATCGTGGCTGTTCGTGGCAT	27128	27109
HAdV-D37_48.9K_R	AAAGCTACAAGAAGAGAGAGCAGCACGA	27600	27573
HAdV-D37_31.6K_R	CTACACTAAGTGTGACTAGGCTAATCA	28918	28892
HAdV-D37_10.4K_R	CGCAGATGTTGAAGAGACAGAAGAGGATGT	29770	29741
HAdV-D37_14.7K_R	AGCATAAGTGGCTAGGGCAGGG	30050	30029
HAdV-D37_14.8K_R	TTGATGGCATCAAGGAGCTCCTT	30497	30475
HAdV-D37_Fiber_R	GAGACAAAGGGGGGAGTGAGGAA	31138	31116
HAdV-D37_i-leader_F	GTGACAAAGAGCCGCTCCGTG	7760	7780
HAdV-D37_i-leader_R	TCACCCGTGGAGATCAAGTACCATCTG	8253	8227
HAdV-D37_j-leader_F	AGGGAACCTACCAGTGCCAGAG	26764	26785
HAdV-D37_j-leader_R	AAGGTTTGATAGTATCAGAAGGAAGGTTAG	26880	26851
HAdV-D37_TPL1_F	ACTCTCTTCCGCGTCGCTGT	5857	5876
HAdV-D37_TPL1_R	CCCAACAGCTGGCGCT	5897	5882
HAdV-D37_TPL2_F	GCGCGGTTGAGGGCGTA	6920	6936
HAdV-D37_TPL2_R	CCGTGCGGACGATCGAG	6991	6975
HAdV-D37_TPL3_F	AAGCGTCTAGCCAATCGCAGTCG	9425	9447
HAdV-D37_TPL3_R	CGACTGCGATTGGCTAGACGCTT	9447	9425
